# Risk of hematological malignancies from CT radiation exposure in children, adolescents and young adults

**DOI:** 10.1038/s41591-023-02620-0

**Published:** 2023-11-09

**Authors:** Magda Bosch de Basea, Isabelle Thierry-Chef, Richard Harbron, Michael Hauptmann, Graham Byrnes, Maria-Odile Bernier, Lucian Le Cornet, Jérémie Dabin, Gilles Ferro, Tore S. Istad, Andreas Jahnen, Choonsik Lee, Carlo Maccia, Françoise Malchair, Hilde Olerud, Steven L. Simon, Jordi Figuerola, Anna Peiro, Hilde Engels, Christoffer Johansen, Maria Blettner, Magnus Kaijser, Kristina Kjaerheim, Amy Berrington de Gonzalez, Neige Journy, Johanna M. Meulepas, Monika Moissonnier, Arvid Nordenskjold, Roman Pokora, Cecile Ronckers, Joachim Schüz, Ausrele Kesminiene, Elisabeth Cardis

**Affiliations:** 1 Barcelona Institute for Global Health (ISGlobal), Barcelona, Spain; 2 Pompeu Fabra University, Barcelona, Spain; 3 Spanish Consortium for Research and Public Health (CIBERESP), Instituto de Salud Carlos III, Madrid, Spain; 4 International Agency for Research on Cancer (IARC/WHO), Environment and Lifestyle Epidemiology Branch, Lyon, France; 5 Population Health Sciences Institute, Newcastle University, Newcastle-upon-Tyne, UK; 6 Institute of Biostatistics and Registry Research, Brandenburg Medical School, Neuruppin, Germany; 7 Institut de Radioprotection et de Sûreté Nucléaire, Fontenay aux Roses, France; 8 Institute of Medical Biostatistics, Epidemiology and Informatics, University Medical Centre of the Johannes Gutenberg University Mainz, Mainz, Germany; 9 German Cancer Research Center, Heidelberg, Germany; 10 Belgian Nuclear Research Centre (SCK CEN), Mol, Belgium; 11 Norwegian Radiation and Nuclear Safety Authority, Oslo, Norway; 12 Luxembourg Institute of Science and Technology, Esch-sur-Alzette, Luxembourg; 13 Division of Cancer Epidemiology and Genetics, National Cancer Institute, National Institutes of Health, Rockville, MD USA; 14 Centre d’Assurance de qualité des Applications Technologiques dans le domaine de la Santé (CAATS), Sèvres, France; 15 Norwegian Radiation Protection Authority, Østerås, Norway; 16 University of South-Eastern Norway, Kongsberg, Norway; 17 Cancer Late Effect Research Oncology Clinic (CASTLE), Center for Surgery and Cancer, Rigshospitalet, Copenhagen, Denmark; 18 Department of Neuroradiology, Karolinska University Hospital, Stockholm, Sweden; 19 Department of Research, Cancer Registry of Norway, Oslo, Norway; 20 Institute of Cancer Research, London, UK; 21 French National Institute of Health and Medical Research (INSERM) Unit 1018, Centre for Research in Epidemiology and Population Health, Paris Saclay University, Gustave Roussy, Villejuif, France; 22 Netherlands Cancer Institute, Amsterdam, the Netherlands

**Keywords:** Risk factors, Epidemiology

## Abstract

Over one million European children undergo computed tomography (CT) scans annually. Although moderate- to high-dose ionizing radiation exposure is an established risk factor for hematological malignancies, risks at CT examination dose levels remain uncertain. Here we followed up a multinational cohort (EPI-CT) of 948,174 individuals who underwent CT examinations before age 22 years in nine European countries. Radiation doses to the active bone marrow were estimated on the basis of body part scanned, patient characteristics, time period and inferred CT technical parameters. We found an association between cumulative dose and risk of all hematological malignancies, with an excess relative risk of 1.96 (95% confidence interval 1.10 to 3.12) per 100 mGy (790 cases). Similar estimates were obtained for lymphoid and myeloid malignancies. Results suggest that for every 10,000 children examined today (mean dose 8 mGy), 1–2 persons are expected to develop a hematological malignancy attributable to radiation exposure in the subsequent 12 years. Our results strengthen the body of evidence of increased cancer risk at low radiation doses and highlight the need for continued justification of pediatric CT examinations and optimization of doses.

## Abstract (web summary)

Analysis of a European cohort estimated that 1–2 per 10,000 children, adolescents and young adults are expected to develop a hematological malignancy within 12 years following computed tomography examination.

## Main

The use of computed tomography (CT) has grown rapidly in most high-income countries^1^ since its introduction^2^ at the beginning of the 1970s. Although the benefits of CT imaging in patient management are undisputed, the potential increased cancer risk^3^ and relatively high cumulative doses incurred from multiple scans have raised concerns in the medical and scientific community, leading to a plateauing/reduction in number of pediatric CTs in many countries^4–6^ and a reduction in pediatric doses^7^. A number of alternative modalities, including fast-acquisition magnetic resonance imaging and ultrasonography are now replacing CT examinations for specific pediatric indications^8^. Despite this, up to 7% of all CT procedures in high-income countries are performed on children^2^.

While moderate-dose (≥100 mGy) to high-dose (≥1 Gy) ionizing radiation exposure is a well-established risk factor for leukemia, in both children and adults^9,10^, the risk associated with childhood and adolescent low-dose exposure (<100 mGy), the dose range typically associated with diagnostic CT examinations, is unclear. This is especially concerning given that CT scanning is the largest contributor to the world’s average annual effective dose per person from medical radiation sources, in both children and adults^2,11^.

Several studies estimated the hematological malignancies risk associated with CT scan radiation in children and young adults in large-scale national cohort^12–18^ and case–control studies^19,20^. Although results of most individual studies^12,13,17,20^ and a recent meta-analysis^21^ suggest an increased risk of leukemia associated with repeated CT examinations, studies were criticized due to low statistical power, inadequate individual dosimetry and potential bias from confounding by indication (when those who undergo CT examinations are at higher risk of cancer than those who do not, due to underlying conditions)^22^. Current international radiological protection recommendations^23^ are, therefore, mainly based on linear extrapolations of risk from the higher doses of the Japanese atomic bomb survivor studies^24^. These extrapolations, which assume no dose threshold below which the risk of radiation-induced cancer is zero (the linear no threshold model of risk), are controversial^10,25^.

The EPI-CT study, coordinated by the International Agency for Research on Cancer (IARC), was set up to overcome limitations of previous national studies and improve direct estimates of cancer risk from low-dose radiation exposure from CT scanning in childhood and adolescence. It included 948,174 individuals from nine European countries^26^. In this Article, we present the EPI-CT analyses of risk of hematological malignancies in relation to radiation exposure from CT examinations in childhood, adolescence and early adulthood.

## Results

### Descriptive analyses

The analysis included 876,771 individuals, who underwent 1,331,896 CT examinations (mean 1.52, standard deviation (s.d.) 1.46 CT examinations per patient) and were followed up for at least 2 years following their first CT. They contributed 6,863,833 person-years (PYs) of follow-up (Table 1). We identified 790 hematological malignancies (subtype distribution in Supplementary Table 1), including 578 cases of lymphoid malignancies and 203 cases of myeloid malignancies and acute leukemia (AL). Mean follow-up was 7.8 years (6.5 years for cases). Fifty-one percent of the cases were younger than 20 years at diagnosis (ranging from 38% among mature T and natural killer (NK) cell neoplasms to 82% among precursor cell neoplasms), whereas 88.5% (range 76–99%) were younger than 30 years (Table 1 and Supplementary Table 2).

**Table 1:** Characteristics of the cohort

	Hematological malignancies—numbers (%)							Entire cohort—*n* (%)	PYs of follow-up—*n* (%)
	All cases	Lymphoid			All myeloid malignancies and AL	Histio. and dendritic cell	Unsp.		
		All^*^	HL	NHL					
Overall	790 (100)	578 (73.2)	190 (24.1)	387 (49.0)	203 (25.7)	6 (0.8)	3 (0.4)	876,771 (100)	6,863,833 (100)
Sex									
Male	466 (59.0)	343 (59.3)	117 (61.6)	226 (58.4)	118 (58.1)	3 (50.0)	2 (66.7)	491,426 (56.0)	3,826,559 (55.7)
Female	324 (41.0)	235 (29.7)	73 (38.4)	161 (41.6)	85 (41.9)	3 (50.0)	1 (33.3)	385,345 (44)	3,037,274 (44.3)
Age at first CT (years)									
<1	93 (11.8)	70 (12.1)	12 (6.3)	58 (15)	22 (10.8)	0 (0)	1 (33.3)	100,628 (11.5)	789,500 (11.5)
1 to <5	126 (15.9)	95 (16.4)	13 (6.8)	82 (21.2)	30 (14.8)	1 (16.7)	0 (0)	149,483 (17.0)	1,159,795 (16.9)
5 to <10	132 (16.7)	98 (17.0)	31 (16.3)	67 (17.3)	33 (16.3)	1 (16.7)	0 (0)	168,135 (19.2)	1,308,483 (19.1)
10 to <15	169 (21.4)	123 (21.3)	58 (30.5)	65 (16.8)	42 (20.7)	3 (50.0)	1 (33.3)	190,561 (21.7)	1,525,680 (22.2)
≥15	270 (34.2)	192 (33.2)	76 (40.0)	115 (29.7)	76 (37.4)	1 (16.7)	1 (33.3)	267,964 (30.6)	2,080,375 (30.3)
Years since first CT examination at end of follow-up									
2 to <5	266 (33.7)	197 (34.1)	55 (28.9)	142 (36.7)	64 (31.5)	4 (66.7)	1 (33.3)	215,041 (24.5)	323,031 (4.7)
5 to <10	263 (33.3)	196 (33.9)	71 (37.4)	124 (32.0)	67 (33.0)	0 (0)	0 (0)	305,667 (34.9)	1,624,031 (23.7)
10 to <15	137 (17.3)	99 (17.1)	42 (22.1)	57 (14.7)	35 (17.2)	2 (33.3)	1 (33.3)	188,762 (21.5)	1,938,588 (28.2)
≥15	124 (15.7)	86 (14.9)	22 (11.6)	64 (16.5)	37 (18.2)	0 (0)	1 (33.3)	167,301 (19.1)	2,978,183 (43.4)
Birth cohort									
<1980	162 (20.5)	115 (19.9)	42 (22.1)	73 (18.9)	45 (22.2)	2 (33.3)	0 (0)	65,725 (7.5)	1,169,822 (17.0)
1980 to <1985	143 (18.1)	94 (16.3)	41 (21.6)	53 (13.7)	47 (23.2)	0 (0)	2 (66.7)	84,747 (9.7)	1,101,016 (16.0)
1985 to <1990	144 (18.2)	108 (18.7)	42 (22.1)	65 (16.8)	35 (17.2)	1 (16.7)	0 (0)	152,209 (17.4)	1,434,265 (20.9)
1990 to <1995	148 (18.7)	113 (19.6)	42 (22.1)	71 (18.3)	34 (16.7)	1 (16.7)	0 (0)	189,513 (21.6)	1,303,426 (19.0)
1995 to <2000	107 (13.5)	81 (14)	17 (8.9)	64 (16.5)	24 (11.8)	1 (16.7)	1 (33.3)	163,306 (18.6)	989,004 (14.4)
2000 to <2005	67 (8.5)	55 (9.5)	6 (3.2)	49 (12.7)	11 (5.4)	1 (16.7)	0 (0)	131,115 (15.0)	643,601 (9.4)
≥2005	19 (2.4)	12 (2.1)	0 (0)	12 (3.1)	7 (3.4)	0 (0)	0 (0)	90,156 (10.3)	222,700 (3.2)
Attained age, years									
2 to <20	404 (51.1)	299 (51.7)	75 (39.5)	224 (57.9)	99 (48.8)	5 (83.3)	1 (33.3)	435,894 (49.7)	2,132,791 (31.1)
20 to <30	295 (37.3)	214 (37)	97 (51.1)	116 (30.0)	78 (38.4)	1 (16.7)	2 (66.7)	320,706 (36.6)	2,752,949 (40.1)
30 to <40	86 (10.9)	62 (10.7)	18 (9.5)	44 (11.4)	24 (11.8)	0 (0)	0 (0)	104,767 (11.9)	1,633,680 (23.8)
≥40	5 (0.6)	3 (0.5)	0 (0)	3 (0.8)	2 (1.0)	0 (0)	0 (0)	15,404 (1.8)	344,414 (5.0)
Country									
Belgium	5 (0.6)	3 (0.5)	0 (0)	3 (0.8)	2 (1.0)	0 (0)	0 (0)	9,052 (1.0)	28,131 (0.4)
Denmark	8 (1.0)	7 (1.2)	2 (1.1)	5 (1.3)	0 (0)	1 (16.7)	0 (0)	15,835 (1.8)	68,053 (1.0)
France	47 (5.9)	43 (7.4)	8 (4.2)	35 (9)	4 (2.0)	0 (0)	0 (0)	104,542 (11.9)	453,713 (6.6)
Germany	23 (2.9)	19 (3.3)	2 (1.1)	17 (4.4)	4 (2.0)	0 (0)	0 (0)	39,501 (4.5)	162,615 (2.4)
The Netherlands	137 (17.3)	98 (17)	30 (15.8)	68 (17.6)	37 (18.2)	2 (33.3)	0 (0)	141,294 (16.1)	1,201,627 (17.5)
Norway	48 (6.1)	36 (6.2)	13 (6.8)	23 (5.9)	11 (5.4)	1 (16.7)	0 (0)	70,942 (8.1)	461,963 (6.7)
Spain	21 (2.7)	17 (2.9)	8 (4.2)	9 (2.3)	4 (2.0)	0 (0)	0 (0)	67,031 (7.6)	253,968 (3.7)
Sweden	107 (13.5)	79 (13.7)	27 (14.2)	51 (13.2)	27 (13.3)	1 (16.7)	0 (0)	119,056 (13.6)	1,151,088 (16.8)
United Kingdom	394 (49.9)	276 (47.8)	100 (52.6)	176 (45.5)	114 (56.2)	1 (16.7)	3 (100)	309,518 (35.3)	3,082,675 (44.9)
Mean bone marrow dose (min–max), mGy									
	20 (0–286)	20 (0–286)	17 (0–209)	22 (0–286)	19 (0–117)	10 (6–22)	19 (13–25)	15.6 (0–1,684)	

Values are shown as number of participants, PYs and mGy. Histio, histiocytic cell malignancies; Unsp, unspecified; min, minimum; max, maximum. *One case could not be classified as HL or NHL.

The distribution of age at first scan was skewed towards later ages, with 30% of the cohort (33% of cases) scanned at age 15 years or above (Table 1 and Supplementary Table 2). This distribution varied by outcome. Among lymphoid malignancies, 70.5% of Hodgkin lymphoma (HL) cases were ≥10 years at the time of their first CT, compared with 46.5% among non-Hodgkin lymphoma (NHL) cases. Among the latter, 62% of mature T and NK cell neoplasm cases were ≥10 years at the time of their first CT compared with 24% precursor cell neoplasm cases (Supplementary Table 2). Among myeloid malignancies and AL cases, the group of myeloproliferative neoplasms (MPNs), myelodysplastic syndrome (MDS) cases and myelodysplastic/myeloproliferative neoplasms (MDS/MPN) also tended to be older at first CT (65% ≥10 years).

About 58% of participants were born between 1985 and 1999 (Table 1). Countries contributed heterogeneously to the EPI-CT cohort (Table 1), with the United Kingdom, the Netherlands, Sweden and France representing 35%, 16%, 14% and 12% of individuals in the cohort, respectively (50%, 17%, 14% and 6% of cases).

The distribution of dose to the active bone marrow (referred to as ABM dose or dose throughout the Article) was strongly positively skewed, with most individuals having received low doses (Extended Data Table 1). The mean and median cumulative ABM dose at end of the follow-up were 15.6 mGy and 10.7 mGy (p25–p75: 5.8–18.2 mGy) (Table 1), respectively, in the cohort and 20 mGy and 13.0 mGy (p25–p75: 6.8–23.2 mGy) among cases overall.

As reported in the previous EPI-CT dosimetry paper^7^, the predominant body part scanned was the head, representing, with neck examinations, approximately 81% of all examinations. For this location, the mean ABM dose decreased by about 25% over the study period in newborns aged 0–3 months (from 15 mGy before 1991 to 12 mGy after 2001) but remained constant in adults aged 17.5 years and older (2.6 mGy). Dose reduction over time was greater for examinations of other body regions: for example, for chest CTs by more than 60% in newborns (from 18 to 7 mGy) and approximately 40% in adults (from 8 to 5 mGy).

### Risk estimation

Elevated relative risks (RRs) for all hematological malignancies combined were observed across all dose categories ≥10 mGy, with a strong dose–response relationship and a RR of 2.66 (95% confidence interval (CI) 1.92 to 3.70) for doses ≥50 mGy compared with doses <5 mGy (reference category) (Table 2). The estimated excess relative risk (ERR) per 100 mGy was 1.96 (95% CI 1.10 to 3.12). Elevated RRs were observed for lymphoid malignancies and for myeloid malignancies and AL separately in most dose categories compared with the reference (Table 2), with risk estimates generally increasing with dose. Continuous risk estimates were very similar for lymphoid malignancies (ERR/100 mGy 2.01, 95% CI 1.02 to 3.42) and myeloid malignancies and AL (ERR/100 mGy 2.02, 95% CI 0.47 to 4.77). The excess absolute risk (EAR) was estimated to be 17.7 per 100,000 PYs per 100 mGy (95% CI 11.6 to 24.0).

**Table 2:** RR and 95% CI per cumulative active bone marrow dose category and ERR/100 mGy by type of hematological malignancy^a^—analyses stratified on sex, birth cohort and country

ABM dose range (mGy)	All hematological malignancies (*n* = 790)				Lymphoid malignancies (*n* = 578)				Myeloid malignancies and AL (*n* = 203)				Leukemia excluding CLL (*n* = 271)			
	#	RR	95% CI		#	RR	95% CI		#	RR	95% CI		#	RR	95% CI	
[0,5)	125	1.00			91	1.00			34	1.00			38	1.00		
[5,10)	171	1.10	0.87	1.39	120	1.07	0.81	1.42	47	1.08	0.69	1.71	43	0.79	0.51	1.24
[10,15)	157	**1.53**	**1.20**	**1.97**	123	**1.65**	**1.24**	**2.20**	32	1.16	0.70	1.92	56	1.35	0.87	2.09
[15,25)	165	**1.40**	**1.09**	**1.80**	121	**1.41**	**1.05**	**1.90**	42	1.31	0.80	2.15	66	1.21	0.78	1.89
[25,50)	114	**1.87**	**1.42**	**2.45**	81	**1.81**	**1.32**	**2.49**	32	**1.96**	**1.17**	**3.29**	44	**1.61**	**1.01**	**2.58**
[50+]	58	**2.66**	**1.92**	**3.70**	42	**2.64**	**1.80**	**3.89**	16	**2.75**	**1.47**	**5.14**	24	**2.41**	**1.40**	**4.17**
*P* for trend		0.02				0.03				0.02				0.02		

	#	ERR/100 mGy	95% CI		#	ERR/100 mGy	95% CI		#	ERR/100 mGy	95% CI		#	ERR/100 mGy	95% CI	
	790	**1.96**	**1.10**	**3.12**	578	**2.01**	**1.02**	**3.42**	203	**2.02**	**0.47**	**4.77**	271	**1.66**	**0.43**	**3.74**

	#	RR at 100 mGy^b^	95% CI		#	RR at 100 mGy	95% CI		#	RR at 100 mGy	95% CI		#	RR at 100 mGy	95% CI	
	790	**2.96**	**2.10**	**4.12**	578	**3.01**	**2.02**	**4.42**	203	**3.02**	**1.47**	**5.77**	271	**2.66**	**1.43**	**4.74**

Values are shown in RR, ERR/100 mGy and 95% CI.

#, number of cases.

Statistically significant values are shown in bold.

^a^No analysis of histiocytic and dendritic cell malignancies or of unspecified malignancies were conducted because of the small number of cases (six and three, respectively).

^b^Note that the RR at 100 mGy is simply obtained by adding 1 to the ERR/100 mGy.

The ERR/100 mGy for NHL was 2.51 (95% CI 1.14 to 4.73) and for HL 1.24 (95% CI 0.08 to 3.28). Increasing trends in RRs with dose were seen for all subtypes (Table 3), although the CIs included unity for mature T and NK cell and for precursor cell neoplasms, and for the MPN + MDS + MDS/MPN grouping. An increased RR compared with the reference dose category was seen at doses as low as 10–15 mGy for NHL as a whole and for mature B cell neoplasms, the largest subgroup. A dose-dependent increase in RR was also seen for leukemia excluding chronic lymphocytic leukemia (CLL) in an analysis using previous classification for comparison with published estimates.

**Table 3:** RR and 95% CI per cumulative active bone marrow dose category and ERR/100 mGy by type of malignancy—analyses stratified on sex, birth cohort and country

a. Lymphoid malignancies other than HL																
ABM dose range (mGy)	Lymphoid malignancies^a^															
	NHL^b^															
	All NHL (*n* = 387)				Mature B cell (*n* = 204)				Mature T and NK cell (*n* = 29)				Precursor cell (*n* = 140)			
	#	RR	95% CI		#	RR	95% CI		#	RR	95% CI		#	RR	95% CI	
[0,5)	53	1.00			32	1.00			7	1.00			13	1.00		
[5,10)	71	1.12	0.78	1.62	48	1.32	0.84	2.09	6	0.86	0.28	2.67	15	0.71	0.33	1.51
[10,15)	85	**1.89**	**1.32**	**2.72**	42	**1.87**	**1.16**	**3.04**	3	0.67	0.17	2.74	35	1.71	0.88	3.35
[15,25)	87	**1.57**	**1.08**	**2.28**	36	1.44	0.86	2.41	7	1.58	0.50	4.95	40	1.31	0.66	2.60
[25,50)	60	**2.08**	**1.40**	**3.10**	29	**2.18**	**1.28**	**3.71**	4	1.66	0.45	6.06	25	1.63	0.79	3.36
[50+]	31	**3.00**	**1.87**	**4.81**	17	**3.63**	**1.95**	**6.76**	2	2.45	0.47	12.71	12	2.10	0.91	4.85
*P* for trend		0.038				0.011				0.046				0.133		

	#	ERR/100 mGy	95% CI		#	ERR/100 mGy	95% CI		#	ERR/100 mGy	95% CI		#	ERR/100 mGy	95% CI	
	387	**2.51**	**1.14**	**4.73**	204	**3.15**	**1.17**	**6.88**	29	2.85	−0.20	20.23	140	1.26	−0.05	4.34

b. HL and myeloid malignancies												
	Lymphoid malignancies^a^				Myeloid malignancies^c^							
ABM dose range (mGy)	HL (*n* = 190)				AML and related precursor neoplasms + ALMP/ALAL (*n* = 80)				MPN + MDS + MDS/MPN (*n* = 115)			
	#	RR	95% CI		#	RR	95% CI		#	RR	95% CI	
[0,5)	38	1.00			13	1			20	1		
[5,10)	49	1.01	0.65	1.55	15	0.81	0.38	1.74	31	1.28	0.72	2.29
[10,15)	38	1.32	0.82	2.10	15	1.18	0.54	2.60	17	1.19	0.61	2.33
[15,25)	34	1.21	0.74	1.98	15	1.01	0.45	2.27	24	1.46	0.76	2.79
[25,50)	20	1.36	0.77	2.38	15	2.01	0.90	4.47	15	1.75	0.86	3.58
[50+]	11	**2.15**	**1.08**	**4.30**	7	2.61	0.99	6.90	8	**2.61**	**1.10**	**6.20**
*P* for trend		0.004				0.04				0.01		

	#	ERR/100 mGy	95% CI		#	ERR/100 mGy	95% CI		#	ERR/100 mGy	95% CI	
	190	**1.24**	**0.08**	**3.28**	80	**2.39**	**0.11**	**8.17**	115	1.51	−0.15	5.06

Values are shown in RR, ERR/100 mGy and 95% CI. Statistically significant values are shown in bold.

^a^One case of lymphoid malignancy could not be classified as HL or NHL—ICD-O-3, 1st revision code 9820.

^b^Fourteen NHL cases could not be classified on the basis of cell type—ICD-O-3, 1st revision code 9590.

^c^Eight cases could not be classified by subgroup: four cases of AL NOS, one acute biphenotypic leukemia and three cases of myeloid leukemia NOS—ICD-O-3, 1st revision codes 9801, 9805 and 9860, respectively.

#, number of hematological malignancy cases.

### Potential confounders of the risk estimates

Removing birth cohort from the model and adjusting for socio-economic status (SES), where available, had little impact on risk estimates (Table 4). Analyses by country (Supplementary Table 3) showed similar numbers of cases of hematological malignancies in the United Kingdom as in the remaining countries combined (394 versus 396). The ERR/100 mGy was about twice as high in the United Kingdom compared with all other countries together, overall (ERR/100 mGy 2.69 versus 1.34), and for lymphoid malignancies and myeloid malignancies and AL separately. Risk estimates varied across countries, particularly for myeloid malignancies and AL, where numbers of cases were low, but estimates were statistically compatible. Analyses removing one country at a time confirmed that only the United Kingdom had a strong influence on the combined risk estimate (Supplementary Tables 3 and 4).

**Table 4:** Effects of potential confounders and potential modifiers of the risk estimates

	All hematological malignancies				Lymphoid malignancies				Myeloid malignancies			
	#	ERR /100 mGy	95% CI		#	ERR /100 mGy	95% CI		#	ERR /100 mGy	95% CI	
Main results^1^	790	**1.96**	**1.10**	**3.12**	578	**2.01**	**1.02**	**3.42**	203	**2.02**	**0.47**	**4.77**
**Potential confounders analysis:**												
No adjustment for birth cohort												
	790	**1.92**	**1.08**	**3.05**	578	**1.99**	**1.02**	**3.37**	203	**1.92**	**0.43**	**4.53**
a) SES^2^												
Unadjusted	210	**1.40**	**0.08**	**3.83**	161	0.99	−0.16	3.37	47	4.22	−0.17	30.6
Adjusted	210	**1.44**	**0.10**	**3.90**	161	1.03	−0.15	3.45	47	4.16	−0.17	29.6
**Effect modification analysis:**												
a) Sex												
Males	466	**1.45**	**0.55**	**2.80**	343	**1.91**	**0.71**	**3.85**	118	0.65	-0.42	2.89
Females	324	**2.82**	**1.27**	**5.32**	235	**2.14**	**0.71**	**4.64**	85	**6.09**	**1.62**	**19.1**
Het. *P* value			0.20				0.85				0.03	
b) Age at exposure category (note: one individual can enter in more than one category if they had several CTs)												
<5	219	**0.78**	**0.06**	**1.78**	165	0.74	−0.05	1.93	52	1.12	−0.29	3.60
5 to <10	156	**1.81**	**0.57**	**3.39**	115	**1.87**	**0.48**	**3.74**	40	1.72	−0.71	5.41
10+	466	**4.02**	**2.48**	**5.99**	336	**4.25**	**2.41**	**6.71**	124	**3.48**	**1.05**	**7.35**
Het. *P* value			0.001				0.002				0.32	
c) Time since exposure (years) (note: one individual can enter in more than one category if they had several CTs)												
2 to <5	303	**3.56**	**1.96**	**5.57**	222	**3.09**	**1.37**	**5.37**	76	**4.88**	**1.66**	**9.87**
5 to <10	291	**2.82**	**1.58**	**4.33**	216	**2.90**	**1.46**	**4.70**	74	**2.98**	**0.66**	**6.40**
10+	260	**1.24**	**0.42**	**2.29**	184	**1.46**	**0.49**	**2.75**	72	0.45	−0.80	2.56
Het. *P* value			0.07				0.21				0.04	

^1^Stratified on sex, birth cohort and country—attained age is used as the underlying time variable.

^2^Analysis restricted to countries where SES data were available: Belgium, France, the Netherlands and Spain.

#, number of cases; Het., heterogeneity.

### Potential modifiers of the risk estimates

There was no evidence of effect modification by sex, except for myeloid malignancies and AL where the elevated ERR was restricted to women (Table 4). The risk increased with increasing age at exposure, especially for lymphoid malignancies, with estimates in the 5–9 and ≥10 years at exposure groups about 2-fold and 3–4-fold those for the <5 years group, respectively. Risk decreased with time since exposure, with risk estimates highest for ABM doses received in the time window ‘2 to <5 years’ and lowest in the time window ‘≥10 years’ before diagnosis. There was, however, no evidence for heterogeneity of risk by time window of exposure, except for myeloid malignancies and AL.

### Sensitivity analyses

Lagging doses by 1 year had little effect on the ERR/100 mGy, while a lag of 5 years reduced the risk by slightly less than half for all and for lymphoid malignancies and by two-thirds for myeloid malignancies and AL (Table 5). Using the median of all dose realizations had no major impact on risk estimates. Substantial ERR increases were noted when excluding individuals with the highest cumulative doses (99th, 98th and 95th percentiles). Excluding 5 and 10 years of follow-up after the first CT increased the estimated ERR/100 mGy for all hematological and for lymphoid malignancies but decreased it for myeloid malignancies and AL; the confidence interval of the latter included zero with a 10-year exclusion (two-thirds of cases excluded). Restricting analyses to individuals born after cancer registration was established in their country/region led to a 10–20% reduction in the ERR/100 mGy depending on outcome, while excluding individuals with a CT in a hospital with low CT reporting consistency had little impact on the risk estimates, except for myeloid malignancies and AL (25% decrease). Restricting the follow-up to 2 years after the maximum age at first CT in each country reduced the number of cases from 790 to 491 and duration of follow-up and resulted in lower, but still elevated, risk estimates particularly for lymphoid malignancies. Excluding individuals with no vital status (*n* = 78,793) slightly reduced risk estimates and increased the width of the CIs, due to the reduction in sample size, for all hematological and lymphoid malignancies, and reduced the myeloid malignancies and AL risk estimates by 31%. Analyses excluding individuals known to have undergone transplantation (United Kingdom only) had little effect on the risk estimate for lymphoid malignancies.

**Table 5:** Results of sensitivity analyses

	All hematological malignancies				Lymphoid malignancies				Myeloid malignancies and AL			
	#	ERR/100 mGy	95% CI		#	ERR/100 mGy	95% CI		#	ERR/100 mGy	95% CI	
Main results	790	1.96	1.10	3.12	578	2.01	1.02	3.42	203	2.02	0.47	4.77
ABM doses												
Doses lagged by 1 year	790	1.99	1.13	3.16	578	2.04	1.05	3.47	203	2.07	0.54	4.80
Doses lagged by 5 years	790	1.06	0.45	1.82	578	1.25	0.53	2.20	203	0.60	−0.43	2.20
Use of median of dose realizations instead of mean	790	2.08	1.09	3.42	578	2.13	0.99	3.75	203	2.17	0.36	5.35
Analyses restricted to individuals with cumulative doses up to:												
99th percentile	777	3.17	1.90	4.91	567	3.02	1.59	5.09	201	3.81	1.43	8.20
98th percentile	761	3.54	2.08	5.55	554	3.27	1.66	5.63	198	4.43	1.67	9.62
95th percentile	710	2.80	1.27	4.94	520	2.79	1.02	5.41	181	2.88	0.34	7.75
Exclusions												
5 years from first CT	524	2.36	1.21	4.05	381	2.54	1.19	4.66	139	1.74	0.05	5.25
10 years from first CT	261	2.67	1.02	5.69	185	3.12	1.13	7.08	72	1.28	-0.62	8.11
Individuals born before the start of cancer registration	490	1.54	0.68	2.80	365	1.59	0.60	3.14	119	1.84	0.20	5.29
Hospitals with low reporting consistency	603	1.93	0.97	3.29	440	2.17	1.00	3.93	158	1.49	0.00	4.31
Follow-up 2 years after country-specific maximum age at exposure	491	1.27	0.49	2.39	356	1.26	0.39	2.63	128	1.61	0.11	6.61
Individuals with no vital status	739	1.73	0.89	2.97	541	1.91	0.91	3.35	189	1.39	0.03	3.85
United Kingdom—main results		NA			276	2.77	1.12	5.55		NA		
Excluding individuals who underwent transplant		NA			256	2.57	0.97	5.31		NA		

#, number of hematological malignancies cases; NA, not applicable.

### Number of CT examinations

An increasing trend in RRs was observed with increasing number of CT examinations (compared with the reference category: one CT examination) both for all hematological and lymphoid malignancies (Supplementary Table 5). In the continuous analyses, risk increased by 43% per examination for hematological malignancies overall, and by 42% and 48%, respectively, for lymphoid and myeloid malignancies and AL.

## Discussion

The EPI-CT study is a large-scale multi-center study designed to directly estimate the risk of hematological malignancies associated with ionizing radiation exposure from CT examinations during childhood and young adulthood, aiming to address criticisms of previous studies related to dosimetry, statistical power and potential biases. The size of the study (nearly one million patients) has considerably increased the statistical power compared with previous national studies. EPI-CT also evaluated risk using the revised World Health Organization (WHO) classification of hematopoietic and lymphoid tissue malignancies^27,28^. Our results showed a clear dose–response between cumulative ABM dose and risk of hematological malignancies, both lymphoid and myeloid, with increased risk at doses as low as 10–15 mGy for NHL as a whole and for mature B cell neoplasms.

Associations between risk of hematological malignancies and estimated CT radiation dose to the active bone marrow were robust to the different assumptions tested in the sensitivity analyses. Risk estimates decreased by about half but remained increased for all hematological and lymphoid malignancies when doses were lagged by 5 years. Risk estimates increased, rather than decreased, when individuals with the highest 1%, 2% and 5% cumulative doses were excluded from the analyses, suggesting they were not unduly affected by outliers.

Prior publications on subsets of the EPI-CT cohort reported higher leukemia risk estimates for national studies in the United Kingdom^12^ and France^16^, but much lower estimates for the Dutch^14^ study compared with the all-countries EPI-CT risk estimates. When applying the EPI-CT dose estimates to the original UK cohort (exposed before 2002 and with follow-up to 2008), using the same classification of leukemia as in the original publications, the ERR/100 mGy was similar to published estimates, though the dose distribution differed somewhat (Supplementary Table 6). Thus, the difference between the EPI-CT risk estimates and the original UK estimates appears attributable to the expanded cohort and longer follow-up (Supplementary Table 6). EPI-CT leukemia risk estimates for France, using the updated French cohort and follow-up^17^, were imprecise due to small numbers of cases in some categories, but compatible with the published French results, even though the dose distribution differed. Differences between the EPI-CT risk estimates and the Dutch data^14^ appear to be mainly related to differences in the dose estimates used, as the results of analyses of the Dutch data using the EPI-CT dosimetry were much closer to those of the full EPI-CT study (Supplementary Table 6). The EPI-CT dosimetry used more sophisticated modeling of doses accounting for historical CT practices and uncertainties due to missing data by country and time period. Final absorbed doses to active bone marrow for each CT examination received were estimated by sex, age group at exposure, body part examined, scanner type and technical scan parameters^7^.

Somewhat surprising was the observation of an increased risk of HL in our analysis, particularly in the light of the absence of an association in the original UK cohort^29^ and the inconsistent results in older adults in other radiation epidemiology studies^30^. Applying the EPI-CT doses to the original UK cohort (with follow-up until 2008) resulted in higher RRs for HL in most dose categories compared with the reference category, with little indication of a dose–response relationship, a different dose distribution (with more individuals receiving higher doses) and a higher ERR/100 mGy (1.1 compared with 0.2), with a CI that included zero (Supplementary Table 6). Analysis of the larger UK EPI-CT cohort, with extended follow-up, yielded an increased ERR/100 mGy (1.73, 95% CI 0.09 to 5.46) suggesting that differences between EPI-CT and published results are mainly attributable to differences in the dosimetry and enlarged cohort size with longer follow-up in the EPI-CT study. While the HL results of the categorical analyses of the full EPI-CT cohort using the UK dose categorization do not show a monotonic trend with dose, analyses using a priori EPI-CT cut points, spanning a wider range of doses, showed evidence of a dose–response (Table 3), with an increased RR in the ≥50 mGy dose category (2.15, 95% CI 1.08 to 4.30). Given the relative rarity of HL compared with NHL, with relatively small numbers of cases in most studies, and in light of the increasing HL incidence in young people, our findings based on 190 cases merit further study.

Within EPI-CT, the UK cohort had a strong influence on risk estimates, contributing about 50% of all hematological malignancies and 45% of the PYs of follow-up. Differences in risk estimates between the United Kingdom and the rest of the countries in the study (also seen for brain tumors)^31^ are unexpected in a multinational collaborative study using a common protocol and dose reconstruction approach. One factor that may partly explain this difference may be the adequacy of the assumptions concerning the technical parameters used during pediatric CT examinations in the United Kingdom, particularly in early years, possibly resulting in a systematic underestimation of doses. Hospital-specific protocols were not available for the United Kingdom^7^, and information from Picture Archiving and Communication System (PACS) data was limited and available only for more recent years. Imaging protocols obtained from pre-existing national surveys in Norway and the United Kingdom had to be used to generate probability density functions (PDFs) of machine settings. These may not adequately reflect the local choices regarding technical parameters made in specific hospitals, particularly in earlier years, which could lead to doses substantially higher than anticipated^32^. Another possible explanation may be related to missing examinations, as the period during which the UK hospitals contributed CT data varied widely between hospitals, contrary to the other countries in the study, and a large proportion of cases were diagnosed in adulthood while only CT examinations up to the age of 22 were included in the study.

EPI-CT was designed to address previous methodological criticisms and limitations of similar studies^12–16^. Reverse causation appears unlikely as risk estimates varied but remained elevated for the major malignancies groupings when greater lags and extended exclusion periods were applied. Neither birth cohort nor SES appeared to confound the associations in the countries where data were available, nor was SES associated with dose in the original UK cohort (A.B.d.G. personal communication).

Despite all efforts, the study presents some limitations. Confounding by indication could not be addressed directly in the full European cohort beyond excluding specific malignancies coded using the International Classification of Diseases for Oncology, 3rd Edition (ICD-O-3) revision 1 as associated with Down syndrome, therapy or organ transplantation, and conducting a sensitivity analysis excluding individuals from the United Kingdom who had undergone organ transplantation. Confounding by indication was, however, evaluated either directly—from a review of medical records—or indirectly—through modeling—in several national EPI-CT cohorts^15–17,33,34^. These analyses support a low likelihood for confounding by indication for leukemia, though it may be more important for lymphoma, as patients are more likely to have immune deficiencies and may be at higher risk of infectious diseases^35^. While appropriate adjustment did not modify radiation-related risk in a recent lymphoma case–control study^35^, the statistical power was low, and the possibility of residual confounding cannot be ruled out.

Information on SES was available in only four of the nine countries (32.3% of the cohort), and the available information covered different SES dimensions, from material deprivation (household income and house value) in the Netherlands to other social determinants of urban vulnerability (including unemployment, unskilled employment and lack of education) in Spain. Adjustment for SES in each country did not materially affect the risk estimates. Residual confounding of the relation between CT radiation dose and risk of hematological malignancies is therefore unlikely to be substantial, particularly since the evidence for an association between different determinants of SES and risk of leukemia (and more generally hematological malignancies) in young people is inconsistent^36^.

While the EPI-CT dose reconstruction is based on sophisticated modeling of doses and associated shared and unshared uncertainties, uncertainties in individual doses are not negligible (geometric s.d. of the order of 2 on average^7^), particularly in early years, and could not be fully integrated in the risk analyses. These uncertainties are unlikely to be differential between cases and non cases. While the shared uncertainties are expected to have little impact on the continuous linear risk estimates, the unshared uncertainties could lead to underestimation of the risk but would not create a spurious association. Further work is needed to validate retrospective dose estimates and to ensure the systematic prospective collection of appropriate dose quantities and technical parameters in the clinic in real time to improve risk estimates in the future.

Unlike in the atomic bomb survivor study^24^, the ERR/100 mGy increased with age at exposure and was highest for exposures within 10 years of diagnosis. These findings, also noted for brain cancers^31^, within EPI-CT, may be an artifact of the generally short follow-up of this cohort (7.8 years on average) and of the heterogeneous distribution of age at exposure, attained age and time since exposure across countries. Further follow-up of this important cohort is needed to increase the statistical power to explore these effects comprehensively.

EPI-CT was conducted to directly estimate risk from CT radiation doses received in childhood, adolescence and young adulthood, avoiding the need for uncertain extrapolations from the atomic bomb survivors and other studies involving higher radiation doses^10,25^. For comparison, our estimates of the ERR/100 mGy in atomic bomb survivors younger than 20 years at exposure were 0.77 (95% CI 0.31 to 1.2) for leukemia excluding CLL, based on 40 cases, and −0.02 (95% CI −99 to 99) for HL, based on 2 cases. Using the revised WHO classification of lymphoid malignancies^37^, the ERR/100 mGy for NHL among those with attained age below 35 was 0.88 (95% CI 0.36 to 3.6), based on small numbers of cases (Ritsu Sakata, personal communication). Numbers were too small to derive risk estimates restricted to survivors exposed below age 20 and for NHL subgroups. The risk estimates for atomic bomb survivors were lower than those in our study. Thus, despite the unavoidable differences in dosimetry systems between the two studies, our results suggest that the linear no threshold model does not overestimate risk from pediatric CT radiation. Indeed, our leukemia risk estimate is compatible with those derived in a recent combined analysis of data on individuals exposed before the age of 21 years and ABM dose <100 mGy (ERR/100 mGy 0.84–4.66, depending on leukemia subtype)^38^.

EPI-CT used the revised WHO classification of lymphoid and myeloid malignancies, which considers cell lineage and different phases of cell differentiation as well as more classical features^27,28^. To our knowledge, the revised classification has only been used in a re-analysis of lymphoma incidence in the atomic bomb survivor cohort^37^. While this classification makes comparisons with previous publications more difficult (we show results for leukemia excluding CLL classification for this purpose), differences in the incidence of different subtypes across populations suggest possible etiological variation, hence possible differences in radiation effects. Indeed, analysis of atomic bomb survivors’ data showed a higher radiation risk of precursor cell NHLs than of mature B or T and NK cell NHLs, contrary to our findings. Differences in length of follow-up and attained age between the atomic bomb survivors’ and the EPI-CT cohorts make any conclusion difficult but emphasize the need for future radiation epidemiological studies to adopt this revised classification.

The analyses presented here showed consistent associations between CT radiation dose and risk of hematological malignancies as a whole, and of lymphoid and myeloid malignancies and AL, with an ERR/100 mGy around 2. With an average ABM dose of 8 mGy for a typical examination today (the average dose in the cohort in 2012–2014), this translates to about a 16% increased risk (95% CI 8% to 24%) of these rare malignancies per examination. In terms of absolute risk, among 10,000 children who receive such an examination today, we expect about 1.4 cases (95% CI 1 to 2) due to CT radiation during the 12 years after the examination.

In conclusion, this large-scale study was designed to directly evaluate cancer risk from pediatric and young adult CT radiation exposure. The results of this study, in which much effort has gone into considering and accounting for possible biases that could affect the risk estimates, strengthen the findings from previous low-dose studies of a consistent and robust dose-related increased risk of radiation-induced hematological malignancies. The findings highlight the need for raising awareness in the medical community and continued strict application of radiological protection measures in medical settings through justification and optimization of radiological procedures, particularly in pediatric populations. This includes ensuring doses are kept as low as reasonably achievable (the ALARA principle), while maintaining appropriate image quality for accurate diagnosis, and monitoring delivered doses; ensuring examinations are justified and unproductive exposure is avoided; and ensuring the benefit-to-risk ratio is maximized for all CT examinations^39^.

## Methods

### Study population

The EPI-CT project set up new cohorts in Belgium, Denmark, the Netherlands, Norway, Spain and Sweden, and included and enlarged existing cohorts in France, Germany and the United Kingdom^12,15,16^. Detailed methods have been published^7,26,40^.

The international EPI-CT cohort includes 948,174 individuals who: (1) underwent at least one CT examination in a participating hospital between 1977 and 2014 before the age of 22 years (exact age limit ranging between 10 and 22 years, depending on country^26^); (2) were residents of geographic areas covered by cancer registries; (3) had no previous history of cancer; and (4) had no cancer diagnosis in the first year following the first CT^40^. In the present analysis we excluded 77,369 individuals with follow-up shorter than 2 years, including 142 individuals with a cancer diagnosis during that period.

The study population was identified through radiology department records of 276 pediatric and general (serving large pediatric patient populations) hospitals. Basic demographic data (including sex, as reported on the clinical history of the patient) and information on each examination was collected for each individual.

### Ethics approvals

The study was approved by the ethics committee at IARC (coordinating center) (IARC IEC 12–35), and the appropriate national, regional and hospital ethics committees in participating countries before starting the epidemiological study. This was a record linkage study with no contact with individual patients (and hence no informed consent).

### Follow-up

Cohort members were followed up through national and/or regional cancer and mortality registries. Germany and part of France lacked information on mortality. Information on migration status was collected where available: in Denmark, Norway and Sweden. In these countries, only 2.05% of cohort members were known to have emigrated during the study follow-up period.

### Outcome definitions

Diagnoses were coded using the International Classification of Diseases for Oncology, 3rd Edition (ICD-O-3) 1st revision (2013). Only cases with behavior code 3 (malignant) were included^41^. Given changes in classification of hematological malignancies according to cell lineage and maturation^27,28^, the analyses were conducted using the revised WHO classification of lymphoid and myeloid malignancies^27,28^, focusing on the following groupings, types and subtypes (morphology codes in Supplementary Table 1):

- All hematological malignancies, excluding those coded as related to therapy or predisposing syndromes as they are unlikely to be related to CT exposure^16,33^;
- All lymphoid malignancies and subgroups of HL, NHL and lymphoid malignancy subtypes (mature B cell, mature T and NK cell, and precursor cell);
- All myeloid malignancies and AL and subgroups of:
  - Acute myeloid leukemia (AML) and related malignancies together with AL of mixed phenotype and ambiguous lineage (ALMP/ALAL);
  - MPN, MDS, together with MDS/MPN—MPN + MDS + MDS/MPN.

For comparison with previous studies, analysis of leukemia, excluding CLL, was also conducted.

### Confounding factors

Information on socioeconomic status (SES) was collected, based on nationally available data sources, in the following countries using the information, for individuals from the following countries, representing 32.3% of the EPI-CT cohort:

- Belgium: SES derived from the healthcare reimbursement classification based on the annual income of the household (two categories: lower or normal);
- France: SES based on Townsend deprivation scores, obtained from linkage of residential postal code (five quintiles) with census data;
- the Netherlands: SES derived from average household income and house value for six-digit postal codes (average population, 40 persons) of cohort members’ residential addresses from Statistics Netherlands;
- Spain: SES based on the Synthetic index of urban vulnerability generated according to the socio-economic characteristics of the census tract that included the area of residence (five quintiles).

No information was available regarding the indication or reasons of the CT examinations.

### Organ dose estimates

The organ dose estimation methodology is described elsewhere^6^. Briefly, it was based on a multi-level approach integrating CT imaging information from hospital questionnaires, national reports, scientific publications, expert opinion together with CT parameter values obtained directly from the PACS from 23% of 276 participating hospitals. Doses were estimated using the National Cancer Institute Dosimetry System for CT^42^. Uncertainty associated with missing parameters, for example, in earlier periods when PACS did not exist, was characterized by a range of possible, realistic values for each missing parameter using the aforementioned sources of information and PDFs defined by age group, sex, body region scanned, machine type representative of technology evolution inferred from questionnaires and time period. For each CT examination, a set of 200 dose realizations was derived where, in each iteration, different values of the parameters were sampled from the PDFs, maintaining proper correlations between parameters.

Our main analyses were based on dose to the active (red) bone marrow (ABM), as commonly used in analyses of hematological malignancies in radiation epidemiology, and the arithmetic mean of all dose realizations for each CT examination. The cumulative dose for each participant was obtained by summing the dose (mean of all realizations) from all examinations the participant received.

### Statistical analysis

Descriptive analyses included the distribution of cases and cohort members by sex, country, age-at-exposure, attained age and time since exposure.

Dose–response analyses were conducted for all outcomes listed above by modeling the RR as 1 + *βΖ*, where *Z* is the cumulative dose and *β* is the ERR per unit dose. The model was fit with proportional hazards regression using the custom-developed R module rERR: Excess Relative Risk Models R package version 0.1 (ref. ^43^). Exact age of the individuals was used as the underlying time variable, and all models were stratified by sex, country and birth cohort (1960–1979, 1980–1984, 1985–1989, 1990–1994, 1995–1999, 2000–2004 and 2005–2012). We also fit an EAR model using the PEANUTS module of the EPICURE software (version 2.00.02) to estimate the absolute excess number of hematological malignancies per 10,000 PYs and per dose *Z*. We used this model to predict the number of cases that would be expected in the European population from CT scanning today as the difference between the total number of cases expected under the fitted model at a typical dose level and the ‘background’ number of cases expected in the absence of radiation exposure.

Analyses used cumulative dose as a continuous variable (in mGy), as well as a categorical variable, with cut points defined on the basis of the cohort dose distribution: 0.0004 to <5, 5 to <10, 10 to <15, 15 to <25, 25 to <50, and 50–1,684 mGy). Due to the skewness of the dose distribution, 95% likelihood-based CIs were used in the continuous analyses. For the categorical analyses, we used Wald-based CI. Trends in RRs by level of dose were tested by fitting the categorical variables as a continuous ordinal variable.

Follow-up started 2 years after the first CT scan (to minimize reverse causation potential) or when complete cancer registration was available in the country/region, whichever was later. Exit date was defined as the earliest of dates of any cancer diagnosis, death, emigration (where available) or end of follow-up in the country/region.

To account for a minimal latency between radiation exposure and malignancy, doses were lagged by 2 years. As EPI-CT is a record linkage study, no information about confounding factors other than birth cohort, sex and country/region was systematically available. The effect of country was assessed in country-specific analyses, and removing one country at a time, and SES effect in analyses restricted to countries with available SES. Effect modification by age at exposure (<5, 5 to <10, and ≥10 years), time since exposure (2 to <5, 5 to <10, and ≥10), sex and birth cohort was tested by including an interaction term with dose in the linear dose model. The statistical significance of model parameters was tested using the likelihood ratio test.

Supplementary and sensitivity analyses were performed to test the findings’ robustness. Regarding doses, analyses included: lagging doses by 1 and 5 years (instead of 2), using the median of all dose realizations instead of the mean, and excluding individuals with the highest cumulative doses (above the 99th, 98th and 95th percentiles of the cumulative dose distribution). Additional analyses were conducted excluding: the first 5 and 10 years of follow-up, individuals born before the start of cancer registration in their respective country/region, and hospitals with low reporting consistency (≥1 consecutive years without reporting CT examinations), as well as excluding individuals from the United Kingdom known to have undergone organ transplantation (transplant data were available only for this country) from the lymphoid malignancies analysis as they are at increased risk of post-transplant lymphoproliferative disorders. We also terminated follow-up 2 years above the age limit for inclusion of scans in each country (as doses received later in life were not collected within the project) and excluded the subcohorts lacking mortality follow-up.

We repeated analyses using the number of CT examinations instead of ABM dose.

To allow comparison of our estimates with those of the atomic bomb survivor study, we conducted analyses of leukemia and HL risk in that study using publicly available grouped incidence data^19^, restricted to the population and follow-up most relevant for EPI-CT: less than 20 years old at time of bombing, with attained age less than 35 years. These analyses, adjusted on attained age, sex, birth cohort and city, were conducted using the AMFIT module of EPICURE.

### Reporting summary

Further information on research design is available in the Nature Portfolio Reporting Summary linked to this article.

## Online content

Any methods, additional references, Nature Portfolio reporting summaries, source data, extended data, supplementary information, acknowledgements, peer review information; details of author contributions and competing interests; and statements of data and code availability are available at 10.1038/s41591-023-02620-0.

## Supplementary information

Supplementary Information Supplementary Tables 1–8.

Reporting Summary

## Figures and Tables

**Extended Data Table 1:**
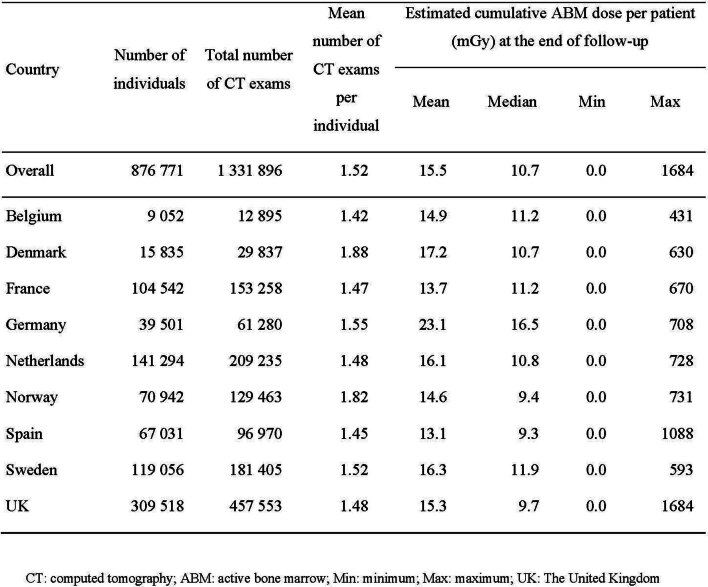
Number of CT examinations and cumulative dose to the active bone marrow per patient, for all individuals and by country

## Data Availability

The data collected and generated in the study are not freely available because of ethical and data protection constraints. The pseudonymized data analysis file for this manuscript is stored at ISGlobal and cannot be shared. Proposals for possible collaborations in further analyses of these data should be addressed to E.C. (elisabeth.cardis@isglobal.org) and will be reviewed by the EPI-CT steering committee. Scientific collaborations will require a written agreement with all involved parties. Requests are normally processed within 1 month. Agreed analysis will be carried out internally by EPI-CT study members, following the agreed scientific collaboration and under the supervision of the proposing researcher. Note that the Data Transfer Agreements (DTAs) ruling the provision of data for the international EPI-CT analyses are time limited and IARC and ISGlobal will be under obligation to destroy the data from individual cohorts when the DTAs expire. Data from these cohorts will be held only by the original data provider, as long as the national data protection legislation permits.

## Code availability

The software used to fit ERR models (the rERR R package) is freely available at https://rdrr.io/cran/rERR/. All EAR models were performed using the PEANUTS module of the EPICURE software (version 2.00.02) commercially available at https://risksciences.com/epicure/. The EAR code applied is available at https://github.com/Mbb2022-23/EPI_CT_EAR’. The ERR code applied is available at https://github.com/radiationISGlobal/EPI_CT_Scripts.

## References

[1] World Bank Country and Lending Groups. *The World Bank* (2023) https://datahelpdesk.worldbank.org/knowledgebase/articles/906519-world-bank-country-and-lending-groups

[2] UNSCEAR 2020/2021 Report Volume I, Scientific Annex A: ‘Medical exposure to ionizing radiation’. *United Nations: Scientific Committee on the Effects of Atomic Radiation.* United Nations, New York (2022) www.unscear.org/unscear/en/publications/2020_2021_1.html

[3] Brenner, D. J. & Hall, E. J. Computed tomography—an increasing source of radiation exposure. *N. Engl. J. Med.* **357**, 2277–2284 (2007). 18046031 10.1056/NEJMra072149

[4] Bosch de Basea, M. et al. Trends and patterns in the use of computed tomography in children and young adults in Catalonia— results from the EPI-CT study. *Pediatr. Radiol.* **46**, 119–129 (2016). 26276264 10.1007/s00247-015-3434-5 PMC4706587

[5] Pearce, M. S. et al. CT scans in young people in Northern England: trends and patterns 1993–2002. *Pediatr. Radiol.* **41**, 832–838 (2011). 21594548 10.1007/s00247-011-2110-7 PMC3992619

[6] Meulepas, J. M. et al. Trends and patterns of computed tomography scan use among children in The Netherlands: 1990–2012. *Eur. Radio.* **27**, 2426–2433 (2017). 10.1007/s00330-016-4566-1 27709278

[7] Thierry-Chef, I. et al. Dose estimation for the European Epidemiological Study on Pediatric Computed Tomography (EPI-CT). *Radiat. Res.* **196**, 74–99 (2021). 33914893 10.1667/RADE-20-00231.1

[8] Marin, J. R. et al. Trends in use of advanced imaging in pediatric emergency departments, 2009–2018. *JAMA Pediatr.* **174**, e202209 (2020). 32761186 10.1001/jamapediatrics.2020.2209 PMC7400208

[9] UNSCEAR 2006 Report. Annex A. Epidemiological studies of radiation and cancer. *United Nations Scientific Committee on the Effects of Atomic Radiation.* United Nations, New York (2006) https://www.unscear.org/unscear/uploads/documents/publications/UNSCEAR_2006_Annex-A-CORR.pdf

[10] Health risks from exposure to low levels of ionizing radiation. BEIR, VII Report, phase II. *Committee on the Biological Effects of Ionizing Radiation*, National Academy Press, Washington DC (2006). http://www.nap.edu/openbook.php?isbn=030909156X

[11] Medical radiation exposure of patients in the United States: recommendations of the National Council on Radiation Protection and Measurements. *NCRP* https://ncrponline.org/shop/reports/report-no-184-medical-radiation-exposure-of-patients-in-the-united-states-2019/ (2019).

[12] Pearce, M. S. et al. Radiation exposure from CT scans in childhood and subsequent risk of leukaemia and brain tumours: a retrospective cohort study. *Lancet* **380**, 499–505 (2012). 22681860 10.1016/S0140-6736(12)60815-0 PMC3418594

[13] Mathews, J. D. et al. Cancer risk in 680,000 people exposed to computed tomography scans in childhood or adolescence: data linkage study of 11 million Australians. *Br. Med. J.* **346**, f2360 (2013). 23694687 10.1136/bmj.f2360 PMC3660619

[14] Meulepas, J. M. et al. Radiation exposure from pediatric CT scans and subsequent cancer risk in the Netherlands. *J. Natl Cancer Inst.* **111**, 256–263 (2019). 30020493 10.1093/jnci/djy104 PMC6657440

[15] Krille, L. et al. Risk of cancer incidence before the age of 15 years after exposure to ionising radiation from computed tomography: results from a German cohort study. *Radiat. Environ. Biophys.* **54**, 1–12 (2015). 25567615 10.1007/s00411-014-0580-3

[16] Journy, N. et al. Are the studies on cancer risk from CT scans biased by indication? Elements of answer from a large-scale cohort study in France. *Br. J. Cancer* **112**, 185–193 (2015). 25314057 10.1038/bjc.2014.526 PMC4453597

[17] Foucault, A. et al. Childhood cancer risks estimates following CT scans: an update of the French CT cohort study. *Eur. Radiol.* **32**, 5491–5498 (2022). 35230516 10.1007/s00330-022-08602-z

[18] Krille, L. et al. Erratum to: Risk of cancer incidence before the age of 15 years after exposure to ionising radiation from computed tomography: results from a German cohort study. *Radiat. Environ. Biophys.* **56**, 293–297 (2017). 28612109 10.1007/s00411-017-0694-5

[19] Li, I.-G., Yang, Y.-H., Li, Y.-T. & Tsai, Y.-H. Paediatric computed tomography and subsequent risk of leukaemia, intracranial malignancy and lymphoma: a nationwide population-based cohort study. *Sci. Rep.* **10**, 7759 (2020). 32385396 10.1038/s41598-020-64805-8 PMC7210298

[20] Nikkilä, A., Raitanen, J., Lohi, O. & Auvinen, A. Radiation exposure from computerized tomography and risk of childhood leukemia: Finnish register-based case–control study of childhood leukemia (FRECCLE). *Haematologica* **103**, 1873–1880 (2018). 29976736 10.3324/haematol.2018.187716 PMC6278981

[21] Berrington de Gonzalez, A., Pasqual, E. & Veiga, L. Epidemiological studies of CT scans and cancer risk: the state of the science. *Br. J. Radiol.* **94**, 20210471 (2021). 34545766 10.1259/bjr.20210471 PMC9328069

[22] Walsh, L., Shore, R., Auvinen, A., Jung, T. & Wakeford, R. Risks from CT scans—what do recent studies tell us? *J. Radiol. Prot.* **34**, E1–E5 (2014). 24594968 10.1088/0952-4746/34/1/E1

[23] The 2007 Recommendations of the International Commission on Radiological Protection. ICRP Publication 103. Ann. *ICRP* 37 (2-4) http://www.icrp.org/publication.asp?id=ICRP%20Publication%20103 (2007). 10.1016/j.icrp.2007.10.003 18082557

[24] Hsu, W.-L. et al. The incidence of leukemia, lymphoma and multiple myeloma among atomic bomb survivors: 1950–2001. *Radiat. Res.* **179**, 361–382 (2013). 23398354 10.1667/RR2892.1 PMC3875218

[25] Tubiana, M. Dose–effect relationship and estimation of the carcinogenic effects of low doses of ionizing radiation: the joint report of the Académie des Sciences (Paris) and of the Académie Nationale de Médecine. *Int. J. Radiat. Oncol. Biol. Phys.* **63**, 317–319 (2005). 16168825 10.1016/j.ijrobp.2005.06.013

[26] Bernier, M.-O. et al. Cohort profile: the EPI-CT study: a European pooled epidemiological study to quantify the risk of radiation-induced cancer from paediatric CT. *Int J. Epidemiol.* **48**, 379–381g (2019). 30388267 10.1093/ije/dyy231 PMC6469297

[27] Arber, D. A. et al. The 2016 revision to the World Health Organization classification of myeloid neoplasms and acute leukemia. *Blood* **127**, 2391–2405 (2016). 27069254 10.1182/blood-2016-03-643544

[28] Swerdlow, S. H. et al. The 2016 revision of the World Health Organization classification of lymphoid neoplasms. *Blood* **127**, 2375–2390 (2016). 26980727 10.1182/blood-2016-01-643569 PMC4874220

[29] Berrington de Gonzalez, A. et al. No association between radiation dose from pediatric CT scans and risk of subsequent Hodgkin lymphoma. *Cancer Epidemiol. Biomark. Prev.* **26**, 804–806 (2017). 10.1158/1055-9965.EPI-16-1011 28052939

[30] Harbron, R. W. & Pasqual, E. Ionising radiation as a risk factor for lymphoma: a review. *J. Radiol. Prot.* **40**, R151–R185 (2020). 10.1088/1361-6498/abbe37 33017815

[31] Hauptmann, M. et al. Brain cancer after radiation exposure from CT examinations of children and young adults: results from the EPI-CT cohort study. *Lancet Oncol.* **24**, 45–53 (2022). 36493793 10.1016/S1470-2045(22)00655-6

[32] Smith-Bindman, R. et al. International variation in radiation dose for computed tomography examinations: prospective cohort study. *Br. Med. J.* **364**, k4931 (2019). 30602590 10.1136/bmj.k4931 PMC6314083

[33] Meulepas, J. M. et al. Confounding of the association between radiation exposure from CT scans and risk of leukemia and brain tumors by cancer susceptibility syndromes. *J. Radiol. Prot.* **36**, 953–974 (2016). 27893452 10.1088/0952-4746/36/4/953

[34] Berrington de Gonzalez, A. et al. Relationship between paediatric CT scans and subsequent risk of leukaemia and brain tumours: assessment of the impact of underlying conditions. *Br. J. Cancer* **114**, 388–394 (2016). 26882064 10.1038/bjc.2015.415 PMC4815765

[35] Pasqual, E. et al. Association of ionizing radiation dose from common medical diagnostic procedures and lymphoma risk in the Epilymph case–control study. *PLoS ONE* **15**, e0235658 (2020). 32649712 10.1371/journal.pone.0235658 PMC7351167

[36] Bosch de Basea, M. et al. CT scan exposure in Spanish children and young adults by socioeconomic status: cross-sectional analysis of cohort data. *PLoS ONE* **13**, e0196449 (2018). 29723272 10.1371/journal.pone.0196449 PMC5933709

[37] Fujihara, M. et al. Incidence of lymphoid neoplasms among atomic bomb survivors by histological subtype, 1950 to 1994. *Blood* **139**, 217–227 (2022). 34428282 10.1182/blood.2020010475 PMC8759532

[38] Little, M. P. et al. Leukaemia and myeloid malignancy among people exposed to low doses (<100 mSv) of ionising radiation during childhood: a pooled analysis of nine historical cohort studies. *Lancet Haematol.* **5**, e346–e358 (2018). 30026010 10.1016/S2352-3026(18)30092-9 PMC6130888

[39] Optimisation of ionising radiation-based medical protocols for diagnostics or therapy. *MEDIRAD* (2022) http://www.medirad-project.eu/recommendations

[40] Bosch de Basea, M. et al. EPI-CT: design, challenges and epidemiological methods of an international study on cancer risk after paediatric and young adult CT. *J. Radiol. Prot.* **35**, 611–628 (2015). 26226081 10.1088/0952-4746/35/3/611

[41] International Classification of Diseases for Oncology, 3rd Edition (ICD-O-3). World Health Organisation, Geneva (2013) http://www.who.int/classifications/icd/adaptations/oncology/en/

[42] Lee, C., Kim, K. P., Bolch, W. E., Moroz, B. E. & Folio, L. NCICT: a computational solution to estimate organ doses for pediatric and adult patients undergoing CT scans. *J. Radiol. Prot.***35**, 891–909 (2015).26609995 10.1088/0952-4746/35/4/891

[43] Badia Roca, F. B. & Moriña Soler, D. rERR: excess relative risk models (2018). https://rdrr.io/cran/rERR/

