# Author Correction: Risk of hematological malignancies from CT radiation exposure in children, adolescents and young adults

**DOI:** 10.1038/s41591-025-03689-5

**Published:** 2025-04-11

**Authors:** Magda Bosch de Basea, Isabelle Thierry-Chef, Richard Harbron, Michael Hauptmann, Graham Byrnes, Maria-Odile Bernier, Lucian Le Cornet, Jérémie Dabin, Gilles Ferro, Tore S. Istad, Andreas Jahnen, Choonsik Lee, Carlo Maccia, Françoise Malchair, Hilde Olerud, Steven L. Simon, Jordi Figuerola, Anna Peiro, Hilde Engels, Christoffer Johansen, Maria Blettner, Magnus Kaijser, Kristina Kjaerheim, Amy Berrington de Gonzalez, Neige Journy, Johanna M. Meulepas, Monika Moissonnier, Arvid Nordenskjold, Roman Pokora, Cecile Ronckers, Joachim Schüz, Ausrele Kesminiene, Elisabeth Cardis

**Affiliations:** 1 Barcelona Institute for Global Health (ISGlobal), Barcelona, Spain; 2 Pompeu Fabra University, Barcelona, Spain; 3 Spanish Consortium for Research and Public Health (CIBERESP), Instituto de Salud Carlos III, Madrid, Spain; 4 International Agency for Research on Cancer (IARC/WHO), Environment and Lifestyle Epidemiology Branch, Lyon, France; 5 Population Health Sciences Institute, Newcastle University, Newcastle-upon-Tyne, UK; 6 Institute of Biostatistics and Registry Research, Brandenburg Medical School, Neuruppin, Germany; 7 Institut de Radioprotection et de Sûreté Nucléaire, Fontenay aux Roses, France; 8 Institute of Medical Biostatistics, Epidemiology and Informatics, University Medical Centre of the Johannes Gutenberg University Mainz, Mainz, Germany; 9 German Cancer Research Center, Heidelberg, Germany; 10 Belgian Nuclear Research Centre (SCK CEN), Mol, Belgium; 11 Norwegian Radiation and Nuclear Safety Authority, Oslo, Norway; 12 Luxembourg Institute of Science and Technology, Esch-sur-Alzette, Luxembourg; 13 Division of Cancer Epidemiology and Genetics, National Cancer Institute, National Institutes of Health, Rockville, MD USA; 14 Centre d’Assurance de qualité des Applications Technologiques dans le domaine de la Santé (CAATS), Sèvres, France; 15 Norwegian Radiation Protection Authority, Østerås, Norway; 16 University of South-Eastern Norway, Kongsberg, Norway; 17 Cancer Late Effect Research Oncology Clinic (CASTLE), Center for Surgery and Cancer, Rigshospitalet, Copenhagen, Denmark; 18 Department of Neuroradiology, Karolinska University Hospital, Stockholm, Sweden; 19 Department of Research, Cancer Registry of Norway, Oslo, Norway; 20 Institute of Cancer Research, London, UK; 21 French National Institute of Health and Medical Research (INSERM) Unit 1018, Centre for Research in Epidemiology and Population Health, Paris Saclay University, Gustave Roussy, Villejuif, France; 22 Netherlands Cancer Institute, Amsterdam, the Netherlands

**Keywords:** Risk factors, Epidemiology

Correction to: *Nature Medicine* 10.1038/s41591-023-02620-0, published online 9 November 2023.

In the version of the article initially published, Magda Bosch de Basea’s surname appeared incorrectly (as Bosch de Basea Gomez) and has now been amended in the HTML and PDF versions of the article.

