# Supplementary material for: Risk of hematological malignancies from CT radiation exposure in children, adolescents and young adults
Source: Nat Med. 2023 Nov 9;29(12):3111–9. doi: 10.1038/s41591-023-02620-0 (PMC10719096; doi:10.1038/s41591-023-02620-0)
Supplement: Supplementary file 1 — Supplementary Tables 1–8. [file 41591_2023_2620_MOESM1_ESM.pdf]

nature medicine

Article https://doi.org/10.1038/s41591-023-02620-0

Risk of hematological malignancies from CT radiation exposure in children, adolescents and young adults

In the format provided by the authors and unedited

Supplementary material

Supplementary Table 1: ICD-O-3 1st revision (WHO, 2013) morphology codes included in the analyses

Main groups[1]	Morphology codes	#
**All haematological malignancies[2]**	9590, 9591, 9596, 9650 - 9653, 9659, 9663, 9665, 9667, 9670, 9671, 9673, 9679, 9680, 9684, 9687, 9690, 9691, 9695, 9698 - 9702, 9708, 9709, 9714, 9718, 9727 - 9729, 9731, 9732, 9735, 9750, 9751, 9754, 9755, 9758, 9761, 9800, 9801, 9805 - 9809, 9811 - 9818, 9820, 9823, 9826, 9827, 9831 - 9837, 9840, 9860, 9861, 9863, 9865 - 9867, 9869 - 9876, 9891, 9895 - 9897, 9910, 9911, 9931, 9940, 9945, 9946, 9948, 9950, 9960 - 9967, 9975, 9980, 9982 - 9986, 9989, 9991, 9992	790
Lymphoid malignancies	9590, 9591, 9596, 9650 - 9653, 9659, 9663, 9665, 9667, 9670, 9671, 9673, 9679, 9680, 9684, 9687, 9690, 9691, 9695, 9698 - 9702, 9708, 9709, 9714, 9718, 9728, 9729, 9731, 9732, 9735, 9761, 9811 - 9818, 9820, 9823, 9826, 9827, 9831 - 9837, 9940, 9948	578
Hodgkin lymphoma	9650 - 9653, 9659, 9663, 9665, 9667	190
Non-Hodgkin lymphoma	9590, 9591, 9596, 9670, 9671, 9673, 9679, 9680, 9684, 9687, 9690, 9691, 9695, 9698 - 9702, 9708, 9709, 9714, 9718, 9728, 9729, 9731, 9732, 9735, 9761, 9811 - 9818, 9823, 9826, 9827, 9831 - 9837, 9940, 9948	387
Mature B-cell neoplasms	9591, 9596, 9670, 9671, 9673, 9679, 9680, 9684, 9687, 9690, 9691, 9695, 9698, 9699, 9731, 9732, 9735, 9761, 9823, 9826, 9833, 9940	204
Mature T-cell or NK-cell neoplasms	9700, 9701, 9702, 9708, 9709, 9714, 9718, 9827, 9831, 9834, 9948	29
Precursor cell neoplasms	9728, 9729, 9811 - 9818, 9835 -9837	140
Myeloid malignancies and acute leukaemia	9727, 9801, 9805 - 9809, 9840, 9860, 9861, 9863, 9865 - 9867, 9869 - 9876, 9891, 9895 - 9898, 9910, 9911, 9920, 9931, 9945, 9946, 9950, 9960 - 9967, 9975, 9980, 9982 - 9987, 9989, 9991, 9992	203
Acute myeloid leukaemia (AML), related precursor malignancies and acute leukaemia of mixed phenotype and ambiguous lineage (ALMP/ALAP)	9727, 9840, 9861, 9865 - 9867, 9869 - 9874, 9891, 9895- 9897, 9910, 9911, 9931	80
Myeloproliferative neoplasms (MPN), Myelodysplastic/myeloproliferative neoplasms (MDS/MPN) together with myelodysplastic syndrome (MDS) - MPN + MDS/MPN + MDS	*9863, 9875,* 9876, 9945, 9946, 9950, 9960-9964, 9975, 9980, 9982-9986, 9989, 9991, 9992	115
Histiocytic and dendritic malignancies	9750, 9751, 9754, 9755, 9758	***6***
Unspecified malignancies	9800	3
Leukaemia excluding CLL (for comparison with previous analyses)	9727, 9801, 9805-9809, 9827, 9835-9837, 9840, 9860, 9861, 9863, 9865-9867, 9869-9876, 9891, 9895-9897, 9910, 9911, 9931, 9940, 9945, 9946, 9948, 9950	271

#Number of cases; CLL: chronic lymphocytic leukaemia

[1] Groups based on the 2016 Updated WHO classification of lymphoid neoplasms (Swerdlow et al 2016) and of myeloid neoplasms and acute leukaemia (Arber et al 2016). All identified cases presented the malignancy behaviour code 3 (fifth digit);

[2] Excluding malignancies related to therapy or predisposing syndromes 9898, 9920, 9971, 9987

Supplementary Table 2: Distribution of study individuals by NHL and Myeloid malignancies and AL subgroups

	Lymphoid malignancies - NHL				Myeloid malignancies and acute leukaemia		
		Mature cells					
	All NHL	B cell neoplasms	T and NK cell neoplasms	Precursor cell neoplasms	All myeloid malignancies and acute leukaemia	AML and related precursor + ALMP/ ALAL*	MPN + MDS/MPN + MDS*
Overall	387	204	29	140	203	80	115
Sex							
Male	226	117	16	86	118	47	66
Female	161	87	13	54	85	33	49
Age at first CT (years)							
<1	58	14	4	39	22	10	11
1-<5	82	38	1	41	30	15	14
5-<10	67	34	6	26	33	14	16
10-<15	65	40	8	14	42	15	26
≥15	115	78	10	20	76	26	48
Years since first examination							
2-<5	142	71	6	61	64	27	35
5-<10	124	57	8	56	67	30	36
10-<15	57	29	6	18	35	12	20
≥15	64	47	9	5	37	11	24
Birth cohort							
<1980	73	49	6	10	45	15	29
1980-<1985	53	40	5	7	47	15	32
1985-<1990	65	32	8	23	35	14	17
1990-<1995	71	39	2	29	34	14	18
1995-<2000	64	21	7	35	24	14	9
2000-<2005	49	18	1	29	11	5	6
≥2005	12	5	0	7	7	3	4

	Lymphoid malignancies - NHL				Myeloid malignancies and acute leukaemia		
		Mature cells					
	All NHL	B cell neoplasms	T and NK cell neoplasms	Precursor cell neoplasms	All myeloid malignancies and acute leukaemia	AML and related precursor + ALMP/ ALAL*	MPN + MDS/MPN + MDS*
Attained age, years							
2-<20	224	93	11	115	99	45	50
20-<30	116	77	11	23	78	29	46
30-<40	44	32	6	2	24	6	17
≥40	3	2	1	0	2	0	2
Country							
Belgium	3	2	0	1	2	0	2
Denmark	5	2	1	2	0	0	0
France	35	11	2	21	4	3	1
Germany	17	7	1	9	4	1	3
The Netherlands	68	36	4	27	37	15	22
Norway	23	7	4	12	11	8	3
Spain	9	5	0	4	4	3	1
Sweden	51	26	3	20	27	8	15
UK	176	108	14	44	114	42	68
Bone marrow dose (range), mGy	22 (0, 286)	21 (1, 286)	22 (2, 196)	23 (0, 243)	19 (0, 117)	20 (0, 95)	18 (0, 117)

Supplementary Table 3: Number of CT scans and cumulative dose to the active bone marrow per individual, for all participants and by country

Country	Number of Individuals	Total number of CT exams	Mean number of CT exams per patient	Estimated cumulative ABM dose per patient (mGy) at the end of follow-up			
				Mean	Median	Min	Max
Overall	876,771	1,331,896	1.52	15.5	10.7	0.0	1684
Belgium	9,052	12,895	1.42	14.9	11.2	0.0	431
Denmark	15,835	29,837	1.88	17.2	10.7	0.0	630
France	104,542	153,258	1.47	13.7	11.2	0.0	670
Germany	39,501	61,280	1.55	23.1	16.5	0.0	708
Netherlands	141,294	209,235	1.48	16.1	10.8	0.0	728
Norway	70,942	129,463	1.82	14.6	9.4	0.0	731
Spain	67,031	96,970	1.45	13.1	9.3	0.0	1088
Sweden	119,056	181,405	1.52	16.3	11.9	0.0	593
UK	309,518	457,553	1.48	15.3	9.7	0.0	1684

CT: computed tomography; ABM: active bone marrow; Min: minimum; Max: maximum; UK: The United Kingdom

Supplementary Table 4: Linear ERR/100 mGy by type of haematological malignancies and by country – adjusted for age, sex, birth cohort - restricted to countries with at least 20 cases

	All haematological malignancies				Lymphoid malignancies				Myeloid malignancies and AL			
	#	ERR/100 mGy	95% CI		#	ERR/100 mGy	95% CI		#	ERR/100 mGy	95% CI	
All countries	*790*	1.96	1.10	3.12	578	2.01	1.02	3.42	203	2.02	0.47	4.77
*By country**												
UK	394	2.69	1.30	4.81	276	2.77	1.12	5.55	114	2.57	0.48	6.93
All but UK	396	1.34	0.41	2.78	302	1.46	0.41	3.19	89	1.21	-0.53	5.67
France	47	0.42	-1.24	6.10	43	0.41	-1.26	6.50	4			
Norway	48	0.63	-0.43	4.51	36	1.37	-0.34	8.74	11			
Sweden	107	1.36	-0.13	4.84	79	2.41	0.18	8.83	27	-1.20	-1.86	2.48
Netherlands	137	1.48	0.00	4.70	98	1.10	-0.20	4.33	37	3.94	-0.57	43.80
Others‡	57	2.85	0.06	14.30	46	1.76	-0.39	11.72	10			
Removing												
Belgium	785	1.93	1.07	3.09	575	1.99	1.01	3.40	201	1.96	0.42	4.69
Denmark	782	1.94	1.08	3.10	571	1.94	0.97	3.34	203	2.02	0.47	4.77
France	743	2.05	1.15	3.29	535	2.13	1.08	3.66	199	2.05	0.48	4.85
Germany	767	1.95	1.07	3.14	559	2.09	1.06	3.58	199	1.76	0.28	4.38
Netherlands	653	2.05	1.10	3.38	480	2.24	1.09	3.94	166	1.81	0.28	4.63
Norway	742	2.10	1.18	3.38	542	2.07	1.02	3.58	192	2.36	0.64	5.49
Spain	769	1.95	1.09	3.13	561	2.02	1.02	3.46	199	1.97	0.44	4.68
Sweden	683	2.06	1.12	3.37	499	1.94	0.90	3.46	176	2.70	0.79	6.29
UK	396	1.34	0.41	2.78	302	1.46	0.41	3.19	89	1.21	-0.53	5.67

AL: acute leukaemia; # Number of haematological malignancy cases | ERR: Excess Relative Risk; 95%CI: 95% confidence intervals;
* Country-specific results presented individually for those with 40 haematological malignancies or more
‡ Others: Belgium, Denmark, Germany and Spain together

Supplementary Table 5: ERR/100 mGy by lymphoid and myeloid malignancy types by country – analyses stratified on sex, birth cohort and country - ***restricted to countries with at least 20 cases***

a. Lymphoid malignancies types

	NHL																HL			
	All NHL				Mature B-cell neoplasms				Mature T- and NK-cell neoplasms				Precursor cell neoplasms							
	#	ERR / 100 mGy	95% CI		#	ERR / 100 mGy	95% CI		#	ERR/ 100 mGy	95% CI		#	ERR/ 100 mGy	95% CI		#	ERR/ 100 mGy	95% CI	
All	387	2.51	1.14	4.73	204	3.15	1.17	6.88	29	2.85	-0.20	20.2	140	1.26	-0.05	4.34	190	1.24	0.08	3.28
Country																				
UK	176	3.73	1.25	8.80	108	4.32	1.28	11.4	14	<0[1]	<0[1]	6.36	44	3.18	-0.56	33.4	100	1.73	0.09	5.46
All but UK	211	1.78	0.45	4.34	96	1.96	0.08	6.93	15	14.19	0.61	Inf	96	0.94	-0.21	4.04	90	0.67	<0[1]	3.60
France	35	-0.47	<0[1]	5.99		NE				NE			21	2.77	<0[1]	Inf		NE		
Norway	23	1.78	-0.38	28.5		NE				NE								NE		
Sweden	51	2.27	-0.05	10.8	26	2.62	-0.34	24.8		NE			20	1.61	-0.62	19.5	27	2.25	-0.85	25.5
Netherlands	68	2.71	0.13	12.1	36	7.71	0.48	Inf		NE			27	0.92	-0.34	8.82	30	<0[1]	<0[1]	1.51
Other	34	1.36	-0.48	12.6		NE				NE								NE		
Removing:																				
Belgium	384	2.48	1.12	4.69	202	3.07	1.14	6.73	29	2.85	-0.20	20.2	139	1.28	-0.04	4.38	190	1.24	0.08	3.28
Denmark	382	2.42	1.07	4.60	202	3.18	1.19	6.96	28	1.84	-0.37	15.0	138	1.23	-0.07	4.27	188	1.22	0.07	3.24
France	352	2.81	1.28	5.36	193	3.80	1.48	8.38	27	2.54	-0.24	17.9	119	1.16	-0.11	4.29	182	1.18	0.03	3.25
Germany	370	2.65	1.19	5.03	197	3.31	1.22	7.30	28	3.08	-0.16	21.8	131	1.28	-0.07	4.56	188	1.31	0.12	3.40
Netherlands·	319	2.47	1.00	4.95	168	2.69	0.82	6.36	25	3.03	-0.19	24.7	113	1.46	-0.21	5.83	160	1.82	0.32	4.62
Norway	364	2.57	1.13	4.93	197	3.26	1.22	7.16	25	0.87	-1.23	13.6	128	1.55	0.04	5.22	177	1.28	0.07	3.50
Spain	378	2.65	1.21	4.99	199	3.28	1.24	7.19	29	2.85	-0.20	20.2	136	1.36	-0.02	4.68	182	1.13	0.02	3.10
Sweden.	336	2.56	1.06	5.09	178	3.25	1.10	7.53	26	2.83	-0.21	20.9	120	1.17	-0.17	4.75	163	1.14	-0.01	3.26

b) Myeloid malignancies and AL types

	AML and related precursor +ALMP/ALAP				MPN + MDS/MPN + MDS*			
	#	ERR/100 mGy	95% CI		#	ERR/100 mGy	95% CI	
All countries	80	2.39	0.11	8.17	115	1.51	-0.15	5.06
*By country**								
UK	42	2.85	-0.09	13.0	68	2.06	-0.13	7.82
All but UK	38	1.75	<0[1]	13.4	47	0.55	<0[1]	6.90
Netherlands	15	6.57	<0[1]	Inf	22	2.10	<0[1]	108.2
Removing								
Belgium	80	2.39	0.11	8.17	113	1.38	-0.23	4.89
Denmark	80	2.39	0.11	8.17	115	1.51	-0.15	5.06
France	77	2.45	0.11	8.51	114	1.52	-0.15	5.10
Germany	79	2.01	-0.14	7.40	112	1.33	-0.25	4.75
Netherlands	65	1.92	-0.18	7.61	93	1.44	-0.23	5.23
Norway	72	2.87	0.27	9.82	112	1.75	-0.06	5.72
Spain	77	2.36	0.09	8.11	114	1.44	-0.18	4.92
Sweden	72	3.14	0.36	10.74	100	2.15	0.09	6.88
UK	38	1.75	-0.73	13.37	47	0.55	<0[1]	6.90

Number of haematological malignancy cases; ERR: Excess Relative Risk; 95%CI: 95% confidence intervals; NE: not estimated as fewer than 20 cases; Inf: Infinite; NHL: non-Hodgkin lymphoma; HL; Hodgkin lymphoma; NK; natural killer; AL: Acute leukaemia; AML; acute myeloid leukaemia; ALMP: cute leukaemia of mixed phenotype; ALAL; acute leukaemia of ambiguous lineage; MPN: myeloproliferative neoplasm; MDS; myelodysplastic syndrome; MDS/MPN: myelodysplastic/myeloproliferative neoplasm

[1]The estimate is on the boundary of the parameter space

Supplementary Table 6: Results of sensitivity analyses

	All haematological malignancies				Lymphoid malignancies				Myeloid malignancies and AL			
	#	ERR/ 100 mGy	95% CI		#	ERR/ 100 mGy	95% CI		#	ERR/ 100 mGy	95% CI	
Main results	790	1.96	1.10	3.12	578	2.01	1.02	3.42	203	2.02	0.47	4.77
ABM doses												
Doses lagged by 1 year	790	1.99	1.13	3.16	578	2.04	1.05	3.47	203	2.07	0.54	4.80
Doses lagged by 5 years	790	1.06	0.45	1.82	578	1.25	0.53	2.20	203	0.60	-0.43	2.20
Use of median of all dose realisations dose instead of mean	790	2.08	1.09	3.42	578	2.13	0.99	3.75	203	2.17	0.36	5.35
Analyses restricted to individuals with cumulative doses up to the:												
99th percentile	777	3.17	1.90	4.91	567	3.02	1.59	5.09	201	3.81	1.43	8.20
98th percentile	761	3.54	2.08	5.55	554	3.27	1.66	5.63	198	4.43	1.67	9.62
95th percentile	710	2.80	1.27	4.94	520	2.79	1.02	5.41	181	2.88	0.34	7.75
Exclusions												
5 years from 1st CT	524	2.36	1.21	4.05	381	2.54	1.19	4.66	139	1.74	0.05	5.25
10 years from 1st CT	261	2.67	1.02	5.69	185	3.12	1.13	7.08	72	1.28	-0.62	8.11
Individuals born before the start of cancer registration	490	1.54	0.68	2.80	365	1.59	0.60	3.14	119	1.84	0.20	5.29
Hospitals with low reporting consistency	603	1.93	0.97	3.29	440	2.17	1.00	3.93	158	1.49	0.00	4.31
Follow-up 2 years after country-specific maximum age at exposure	491	1.27	0.49	2.39	356	1.26	0.39	2.63	128	1.61	0.11	6.61
Individuals with no vital status	739	1.73	0.89	2.97	541	1.91	0.91	3.35	189	1.39	0.03	3.85
UK - main results		NA			276	2.77	1.12	5.55		NA		
Excluding individuals who underwent transplant		NA			256	2.57	0.97	5.31		NA		

Number of haematological malignancies cases; ERR: Excess Relative Risk; 95%CI: 95% confidence intervals; NA: not applicable; AL: Acute leukaemia

Supplementary Table 7: RR and 95% CI by category of number of CT examinations and ERR/ per examination for haematological malignancies– analyses stratified on sex, birth cohort and country

	All haematological malignancies				Lymphoid malignancies				Myeloid malignancies and AL			
Number of examinations	#	RR	95% CI		#	RR	95% CI		#	RR	95% CI	
1	482	1.00			355	1.00			122	1.00		
2-3	195	1.53	1.30	1.81	132	1.39	1.13	1.70	59	1.95	1.43	2.66
4-5	66	2.05	1.48	2.84	53	2.32	1.62	3.32	13	1.32	0.58	3.00
6+	47	2.84	1.99	4.04	38	2.90	1.93	4.35	9	2.80	1.37	5.74
P for trend		*0.12*				*0.18*				*0.12*		
	#	ERR/ exam	95% CI		#	ERR/ exam	95% CI		#	ERR/ exam	95% CI	
	790	0.43	0.25	0.73	579	0.42	0.22	0.77	203	0.48	0.13	1.51

Number of haematological malignancies cases; RR: Relative Risk; 95%CI: 95% confidence intervals; ERR: Excess Relative Risk; exam: examination; AL: Acute leukaemia

Supplementary Table 8: Relative risks (and 95% CIs) comparison with previously published results· Risk estimates are reported as per the original studies and as recalculated with the EPI-CT dose estimates

	Dosimetry	RR<br>#	RR (95% CI)<br>#			RR (95% CI)<br>#			RR (95% CI)<br>#			RR (95% CI)<br>#			RR (95% CI)<br>#			ERR/100 mGy<br>(95% CI) #		
Leukaemia excluding CLL																				
**All countries**	***Doses (mGy)***	***[0,5)***	***[5,10)***			***[10,15)***			***[15,25)***			***[25,50)***			***50+***					
EPI- CT all countries	EPI-CT	**1.0**	**0.79**	0.51	1.24	**1.35**	0.87	2.09	**1.21**	0.78	1.89	**1.61**	1.01	2.58	**2.41**	1.40	4.17	**1.66**	0.43	3.74
		38	43			56			66			44			24			271		
**UK definitions**	***Doses (mGy)***	***[0,5)***	***[5,10)***			***[10,15)***			***[15,20)***			***[20,30)***			***30+***					
Leukaemia excluding CLL and including MDS[1]																				
EPI- CT all countries	EPI-CT	**1.0**	**1.00**	0.67	1.47	**1.46**	0.98	2.16	**1.49**	0.96	2.30	**1.37**	0.89	2.11	**2.36**	1.57	3.56	**1.88**	0.65	3.87
		46	62			67			48			54			67			344		
UK – EPI-CT cohort and follow-up<br>*309 518 individuals followed-up to 2013*	EPI-CT	**1.0**	**1.01**	0.59	1.73	**1.55**	0.88	2.72	**1.70**	0.93	3.13	**1.28**	0.68	2.40	**2.82**	1.61	4.96	**2.77**	0.72	6.85
		26	29			27			24			21			34			161		
UK – Original study (Pearce et al, 2012)<br>*178 604 individuals followed-up to 2008*	EPI-CT	**1**	**1.93**	0.72	5.20	**4.25**	1.61	11.2	**4.23**	1.48	12.1	**3.23**	1.11	9.39	**4.94**	1.74	140	**3.78**	0.18	17.5
		6	13			17			13			12			13			74		
	Original	**1**	**1.44**	0.70	2.99	**2.03**	0.89	4.54	**1.53**	0.63	3.59	**2.02**	0.56	5.83	**3.18**	1.46	6.94	**3.60**	0.50	12.0
		15	17			12			11			4			15			74		
**FRANCE definitions**	***Doses (mGy)***	***[0,5)***	***[5,10)***			***[10,19)***			***[20+)***											
Leukaemia excluding CLL and including MDS[1] - CTs up to age 10 and follow-up to age 16 years																				
France - EPI-CT cohort and follow-up<br>*104542 individuals followed-up till 2013*	EPI-CT	**1.0**	**0.58**	0.10	3.49	**1.75**	0.50	6.16	**2.00**	0.47	8.53							**3.12**	-1.4	175
		3	2			15			5									24		
France – original study (Journy et al, 2015) - *67 274 individuals followed-up till 2010*	Original	**1.0**	**1.36**	0.39	4.54	**1.00**	0.21	3.80	**3.14**	0.65	12.2	**NA**						**4.7**	-1	19
		6	5			3			3									17		

	Dosimetry	RR #	RR (95% CI) #			RR (95% CI) #			RR (95% CI) #			RR (95% CI) #			RR (95% CI) #			ERR/100 mGy (95% CI) #		
**Netherlands definitions**	***Doses (mGy)***	***[0,5)***	***[5,10)***			***[10,17)***			***[17+)***											
Leukaemia with myelodysplastic diseases[1]																				
EPI- CT all countries	EPI-CT	**1.00**	**0.96**	0.65	1.42	**1.38**	0.94	2.01	**1.76**	1.22	2.53							**1.79**	0.60	3.71
		48	62			84			153									347		
**Netherlands** – original study (Meulepas et al, 2019)	EPI-CT	**1.0**	**0.94**	0.34	2.64	**1.83**	0.69	4.85	**2.45**	0.96	6.24							**2.44**	-0.05	12.2
		6	10			15			31									62		
	Original	**1.0**	**1.41**	0.7	2.83	**0.88**	0.44	1.74	**0.62**	0.28	1.34							**0.04**	-0.1	1.61
		20	14			16			13									63		
Hodgkin lymphoma																				
	Doses (mGy)	***[0,5)***	***[5,10)***			***[10,15)***			***[15,20)***			***[20+)***								
**EPI- CT all countries**	EPI-CT	**1.0**	**1.01**	0.65	1.56	**1.31**	0.82	2.10	**0.86**	0.46	1.63	**1.61**	1.03	2.52				**1.24**	0.08	3.28
		38	49			38			14			51						190		
**UK** – EPI-CT cohort and follow-up	EPI-CT	**1.0**	**1.39**	0.78	2.48	**1.48**	0.76	2.90	**1.15**	0.49	2.70	**1.72**	0.91	3.24				**1.73**	0.09	5.46
		20	30			17			8			25						100		
**UK** – Original study (Berrington de Gonzales et al, 2017)	EPI-CT	**1.0**	**1.74**	0.82	3.70	**1.54**	0.62	3.78	**0.82**	0.21	3.10	**1.67**	0.68	4.10	**NA**			**1.10**	-0.5	6.2
		11	21			10			3			12						57		
	Original	**1.0**	**1.00**	0.54	1.86	**0.79**	0.31	1.99	**1.05**	0.41	2.68	**0.92**	0.38	2.22	NA			**0.2**	-1.6	2.1
		27	18			6			7			7						65		

RR: Relative Risk; ERR: Excess Relative Risk; CLL: chronic lymphocytic leukaemia; #: Number of cases; NA: not available; *Reference category

[1]ICDO-3 rev 1 morphology included (only tumours with behaviour code 3 (malignant))

- UK (Pearce et al 2012, Supplementary table 1): 9727-9729, 9800, 9801, 9805, 9820, 9835-9837, 9840, 9860, 9861, 9863, 9866, 9867, 9870-9874, 9875, 9876, 9891, 9895- 9897, 9910, 9920, 9930, 9931, 9945, 9946, 9950, 9960-9964, 9975, 9980, 9982-9987, 9989
- France (Journy et al 2015, Supplementary Table 1): 9836, 9837, 9866, 9897, 9910, 9946, 9826, 9983
- Netherlands (Meulepas et al 2018, Supplementary Table 3) : 9727-9729, 9800, 9801, 9805-9809, 9823, 9835-9837, 9840, 9860, 9861, 9863, 9866, 9867, 9870-9874, 9875, 9876, 9891, 9895-9897, 9910, 9920, 9931, 9945, 9946, 9950, 9960-9964, 9965-67, 9975, 9980, 9982-9986, 9987, 9989, 9991, 9992

References

Arber, D. A. *et al.* The 2016 revision to the World Health Organization classification of myeloid neoplasms and acute leukemia. *Blood* **127**, 2391–2405 (2016).

Berrington de Gonzalez, A. *et al.* No Association between Radiation Dose from Pediatric CT Scans and Risk of Subsequent Hodgkin Lymphoma. *Cancer Epidemiol Biomarkers Prev* **26**, 804–806 (2017).

Journy, N. *et al.* Are the studies on cancer risk from CT scans biased by indication? Elements of answer from a large-scale cohort study in France. *Br J Cancer* **112**, 185–193 (2015).

Meulepas, J. M. *et al.* Radiation Exposure From Pediatric CT Scans and Subsequent Cancer Risk in the Netherlands. *J Natl Cancer Inst* **111**, 256–263 (2019).

Pearce, M. S. *et al.* Radiation exposure from CT scans in childhood and subsequent risk of leukaemia and brain tumours: a retrospective cohort study. *Lancet* **380**, 499–505 (2012).

Swerdlow, S. H. *et al.* The 2016 revision of the World Health Organization classification of lymphoid neoplasms. *Blood* **127**, 2375–2390 (2016).

WHO | International Classification of Diseases for Oncology, 3rd Edition (ICD-O-3), 1st revision. *WHO* http://www.who.int/classifications/icd/adaptations/oncology/en/ (2013)
