# Supplementary material for: Risk of hematological malignancies from CT radiation exposure in children, adolescents and young adults
Source: Nat Med. 2023 Nov 9;29(12):3111–9. doi: 10.1038/s41591-023-02620-0 (PMC10719096; doi:10.1038/s41591-023-02620-0)
Supplement: Supplementary file 2 — Reporting Summary [file 41591_2023_2620_MOESM2_ESM.pdf]

nature portfolio

Corresponding author(s): Cardis Elisabeth

Last updated by author(s): Sep 21, 2023

Reporting Summary

Nature Portfolio wishes to improve the reproducibility of the work that we publish. This form provides structure for consistency and transparency in reporting. For further information on Nature Portfolio policies, see our Editorial Policies and the Editorial Policy Checklist.

Statistics

For all statistical analyses, confirm that the following items are present in the figure legend, table legend, main text, or Methods section.

n/a	Confirmed	
☒	☐	The exact sample size (*n*) for each experimental group/condition, given as a discrete number and unit of measurement
☒	☐	A statement on whether measurements were taken from distinct samples or whether the same sample was measured repeatedly
☐	☒	The statistical test(s) used AND whether they are one- or two-sided<br>*Only common tests should be described solely by name; describe more complex techniques in the Methods section.*
☐	☒	A description of all covariates tested
☐	☒	A description of any assumptions or corrections, such as tests of normality and adjustment for multiple comparisons
☐	☒	A full description of the statistical parameters including central tendency (e.g. means) or other basic estimates (e.g. regression coefficient) AND variation (e.g. standard deviation) or associated estimates of uncertainty (e.g. confidence intervals)
☐	☒	For null hypothesis testing, the test statistic (e.g. *F*, *t*, *r*) with confidence intervals, effect sizes, degrees of freedom and *P* value noted<br>*Give P values as exact values whenever suitable.*
☒	☐	For Bayesian analysis, information on the choice of priors and Markov chain Monte Carlo settings
☒	☐	For hierarchical and complex designs, identification of the appropriate level for tests and full reporting of outcomes
☐	☒	Estimates of effect sizes (e.g. Cohen's *d*, Pearson's *r*), indicating how they were calculated

Our web collection on statistics for biologists contains articles on many of the points above.

Software and code

Policy information about availability of computer code

Data collection	*Provide a description of all commercial, open source and custom code used to collect the data in this study, specifying the version used OR state that no software was used.*
Data analysis	R (latest 4.30), EPICURE (version 2.00.02), rERR: Excess Relative Risk Models R package version 0.1<br>Codes developed are available in GitHub https://github.com/Mbb2022-23/EPI_CT_EAR", https://github.com/radiationISGlobal/EPI_CT_Scripts

For manuscripts utilizing custom algorithms or software that are central to the research but not yet described in published literature, software must be made available to editors and reviewers. We strongly encourage code deposition in a community repository (e.g. GitHub). See the Nature Portfolio guidelines for submitting code & software for further information.

Data

Policy information about availability of data

All manuscripts must include a data availability statement. This statement should provide the following information, where applicable:

- Accession codes, unique identifiers, or web links for publicly available datasets
- A description of any restrictions on data availability
- For clinical datasets or third party data, please ensure that the statement adheres to our policy

The data collected and generated in the study are not freely available because of ethical and data protection constraints. The pseudonymized data analysis file for this manuscript is stored at ISGlobal and cannot be shared. Proposals for possible collaborations in further analyses of these data should be addressed to Professor

Elisabeth Cardis (elisabeth.cardis@isglobal.org) and will be reviewed by the EPI-CT steering committee. Scientific collaborations will require a written agreement with all involved parties. Requests are normally processed within 1 month. Agreed analysis will be carried out internally by EPI-CT study members, following the agreed scientific collaboration and under the supervision of the proposing researcher. Note that the Data Transfer Agreements (DTA) ruling the provision of data for the international EPI-CT analyses are time limited and IARC and ISGlobal will be under obligation to destroy the data from individual cohorts when the DTAs expire. Data from these cohorts will be held only by the original data provider, as long as the national data protection legislation permits.

Research involving human participants, their data, or biological material

Policy information about studies with human participants or human data. See also policy information about sex, gender (identity/presentation), and sexual orientation and race, ethnicity and racism.

Reporting on sex and gender	All analyses include both females and males and are adjusted for sex through stratification.<br>We also conducted an analysis to evaluate whether the risk might differ between males and females. There was no evidence for a difference of risk between sexes overall though the risk of myeloid malignancies and AL appeared to be higher in women than men.<br>No data was available on gender in this study
Reporting on race, ethnicity, or other socially relevant groupings	Race and ethnicity information was not available in this study.<br>Information on socio-economic status (SES) was collected, based on nationally available data sources, in the following countries using the information, available for study subjects from the following countries, representing 32.3% of the EPI-CT cohort:<br>• Belgium: SES derived from the healthcare reimbursement classification based on the annual income of the household (2 categories: lower or normal);<br>• France: SES based on Townsend deprivation scores, obtained from linkage of residential postal code (5 quintiles) with census data·<br>• the Netherlands: SES derived from average household income and house value for six-digit postal codes (average population, 40 persons) of cohort members' residential addresses from Statistics Netherlands;<br>• Spain: SES based on the Synthetic index of urban vulnerability generated according to the socioeconomic characteristics of the census tract that included the area of residence (5 quintiles).<br>This variable was used to evaluate whether the relation between radiation dose from CT scans and risk of haematological malignancies might be confounded by SES and to adjust for this if this is the case. The reason SES might be a confounder is that on one side, SES and urban vulnerbaility or deprivation could be related to the likelihood of undergoing a CT scan (because of trauma for example) and, on the other side SES has been suggested to be related to the risk of developing leukaemia though the evidence is not conclusive.<br>Potential confounding was evaluated by conducting analyses of risk restricted to the four countries where SES Information was available including and excluding SES as a covariate in the model and checking whether inclusion of SES modified risk estimates by at least 10%.
Population characteristics	The study population includes 948,174 subjects (males and females aged 0 to over 50 years old) who: 1) underwent at least one CT examination in a participating hospital between 1977 and 2014 before the age of 22 years, 2) had no previous history of cancer, and 3) had no cancer diagnosis in the two years following the 1st CT
Recruitment	The study population was identified through radiology department records of 276 paediatric and general (serving large paediatric patient populations) hospitals in the study regions. The population includes all partients in these services who meet the criteria described above under Population Characteristics.<br>This is an entirely record based study and hence is not subject to participation bias.<br>A few hospitals did not provide data for the entire study period thus possibly leading to underestimation of doses for some patients (see discussion).<br>The hospitals do not cover all of the hospitals in the study regions hence there is the potential for missing CT examinations. However the participating hospitals are expected to cover the vast majority of the CT scans in this population in the study region since the study was based on the specialised paediatric hospitals and the large general hospitals with large paediatric populations.
Ethics oversight	IARC Ethics Committee (IARC IEC 12–35) and the appropriate national, regional and hospital ethics committees in participating countries (the study included participation of 276 radiology departments from 9 countries,in addition to the various PI institutions and national cancer and population registries)

Note that full information on the approval of the study protocol must also be provided in the manuscript.

Field-specific reporting

Please select the one below that is the best fit for your research. If you are not sure, read the appropriate sections before making your selection.

☒ Life sciences ☐ Behavioural & social sciences ☐ Ecological, evolutionary & environmental sciences

For a reference copy of the document with all sections, see nature.com/documents/nr-reporting-summary-flat.pdf

Life sciences study design

All studies must disclose on these points even when the disclosure is negative.

Sample size	Statistical power was evaluated in a feasibility study in the EC funded CHILD-MED-RAD project, before the launch of the EPI-CT study. It was estimated based on the expected number of subjects that could be included in each country, the expected duration of follow-up and risk estimates of radiation induced cancer risk by age at exposure and time from the latest follow-up of the atomic bomb survivors study. Calculations were made using the US NIH "Power software" http://dceg.cancer.gov/tools/design/power) based on the following assumptions. Distributions of numbers of scans and body part scanned are based on data collected to date in the previously published UK study (Pearce et al 2012).<br>The specific assumptions for leukaemia analyses were as follows:<br>• 7% non-exposed as doses to red bone marrow from head scans appear similar (about 5 mGy) to those to red bone marrow in the spine from chest and abdominal CTs – hence only extremity scans would be assumed to be non-exposed<br>• 5 mGy to the red bone marrow per scan<br>• RRs of the order of 1.75, 2, and 2.5 associated with 10 scans (i.e. 50 mGy) in the first 10 years of follow-up<br>• an average incidence rate of 5 per 100 000 per year (based on Spanish figures in this age range<br>• an average follow-up time of about 11 years<br>• and hence the probability of developing a disease would be 5.6 per 10 000 for each person<br>• about 2% of the paediatric population undergoing CT every year.<br>For a RR of 2.5 associated with 10 CT scans (ie 50 mGy) in the first 10 years of follow-up, we expected 80% power with a cohort of 500 000 patients. For a RR of 1.75 we needed a cohort of 1.2 Million patients to reach 80% power.
Data exclusions	We excluded from follow-up the first 2 years after the first CT examination to minimize reverse causation potential as well as the years when complete cancer registration was not available in the subject's country/region<br>We also excluded from analyses of haematological malignancies those coded as related to therapy or predisposing syndromes as they are unlikely to be related to CT exposure
Replication	This is a very large scale epidemiological study and no replication was logistically feasible.<br>However, to ensure the validity of the results, analyses were conducted by country/group of countries to ensure that no single country drove the results and conclusion of the study.<br>We also conducted numerous sensitivity analyses to address potential biases which could affect the interpretation of the results.<br>We compared our results to those of other similar studies and to those of other studies of low to moderate doses of ionising radiation in childhood and adolescence (see discussion)
Randomization	Not applicable as this was an observational study
Blinding	The investigators who extracted the information from the radiological records and who estimated the radiation doses were blinded to the cancer and mortality status of the study subjects.

Reporting for specific materials, systems and methods

We require information from authors about some types of materials, experimental systems and methods used in many studies. Here, indicate whether each material, system or method listed is relevant to your study. If you are not sure if a list item applies to your research, read the appropriate section before selecting a response.

Materials & experimental systems

n/a	Involved in the study	
☒	☐	Antibodies
☒	☐	Eukaryotic cell lines
☒	☐	Palaeontology and archaeology
☒	☐	Animals and other organisms
☒	☐	Clinical data
☒	☐	Dual use research of concern
☒	☐	Plants

Methods

n/a	Involved in the study	
☒	☐	ChIP-seq
☒	☐	Flow cytometry
☒	☐	MRI-based neuroimaging
